# *In Silico* and Biochemical Characterization of Lysozyme-Like Proteins in the Rat

**DOI:** 10.1371/journal.pone.0161909

**Published:** 2016-09-09

**Authors:** Ganapathy Narmadha, Suresh Yenugu

**Affiliations:** Department of Animal Biology, University of Hyderabad, Hyderabad—500 046, India; National Cancer Institute, UNITED STATES

## Abstract

**Background:**

Spermatogenesis and sperm maturation in the male reproductive tract is dictated by a variety of proteins secreted in the testis and epididymis. Though the proteome of these tissues is known, the functional role of many of these proteins remains uncharacterized. In this study, we characterize the rat Lysozyme-like (*Lyzl*) genes and proteins.

**Methods:**

*In silico* tools were used to predict the primary, secondary and tertiary structures. Reverse transcription PCR, immunofluorescence and immunoblotting were used to determine the expression pattern. Lysozyme like enzyme activity was assessed by standard assays.

**Results:**

Six rat *Lyzl* genes namely *Lyzl1*, *Lyzl3*, *Lyzl4*, *Lyzl5*, *Lyzl6* and *Lyzl7* were found to be highly conserved among the vertebrates with higher homology to mouse counterparts than with human counterparts. All the LYZL proteins contained the characteristic 4 disulfide bridges similar to c-type lysozyme. Only LYZL 1 and 6, conserved the active site amino acids of the lysozyme. Molecular modeling studies indicated that LYZL proteins exhibit strikingly similar three-dimensional structures among themselves. The secondary structure analysis of the recombinant LYZL proteins indicated the presence of α-helix, β-sheet and random coil with α-helix being the majority. Docking studies indicated the peptidoglycan binding nature of LYZL proteins. All the rat *Lyzl* mRNA transcripts (*Lyzl1*, *Lyzl3*, *Lyzl4*, *Lyzl5*, *Lyzl6* and *Lyzl7)* are predominantly expressed in testes though some of them are expressed in tissues other than reproductive tract. Their expression was androgen independent. The rat LYZL proteins are localized in the germinal epithelium and on the spermatozoa. Recombinant LYZL1 and 6 possessed muramidase, isopeptidase and antibacterial activities. The mechanism of antibacterial action of LYZL1 and LYZL6 involved bacterial membrane damage and leakage of cellular contents. Only LYZL1 and 6 possess peptidoglycan binding ability, whereas LYZL3, LYZL4 and LYZL5 possess hyaluronan binding ability suggesting a possible functional divergence of these proteins. LYZL3, LYZL4 and LYZL7 possessed free radical scavenging property, suggesting that they may act as antioxidants.

**Conclusion:**

The divergent properties of LYZL proteins indicate that they may have a role in sperm function, innate immunity and other physiological process as well.

## Introduction

Testicular and epididymal secretions aid the maturation of mammalian spermatozoa to acquire fertilizing ability and this process that involves a series of complex and sequential events involving structural, physiological and biochemical changes. A comparison of the proteomes of testes, epididymis and spermatozoa revealed that 47% of the proteins in the sperm are intrinsic and are acquired from testes [1]. 23% of the proteins are extrinsic that are acquired from the environment, clearly suggesting that secretory proteins in the lumen are added on to the sperm surface. It is also reported that acrosomal protein content of caput and caudal sperm are different, suggesting that sperm undergoes changes during the transit and this is due to addition of a wide variety of proteins added on to its surface. Examples include HongrES1 [2], HE4 [3], cystatin 11 (CST11) [4], lactoferrin [5], human cathelicidin antimicrobial peptide (hCAP18) [6], ESP13.2 [7], members of the SPAG11 family [8], members of the PATE family [9] and defensins [10]. Some of the members of defensin, SPAG11 and PATE families are shown to have role in fertilization, suggesting bifunctional role for these proteins in epididymal innate immunity and sperm maturation [7,9,11]. Further, amyloidogenic proteins such as cystatin-related epididymal spermatogenic (CRES) protein in the acrosomal matrix of the spermatozoa form amyloids *in vitro* and *in vivo*, besides being antimicrobial [12] [13] [14]. Thus, proteins secreted on to the sperm surface exert diverse function and have been drawing attention in the last two decades.

Among the different types of lysozymes, namely, g-type (goose-type), i-type (invertebrate-type), c-type (chicken or conventional-type), plant, phage and bacterial, the c-type is the predominant type and widely expressed in many species. A common feature of most lysozymes is that they possess antibacterial activity [15]. Recently lysozyme- like (*Lyzl*) genes that belong to c-type lysozyme family were identified. Zhang et al., reported the mRNA expression of *Lyzl*2, *Lyzl3*, *Lyzl4* and *Lyzl6* in humans tissues [16]. *Lyzl*2, *Lyzl4* and *Lyzl6* mRNA were found to be expressed only in the testes and *Lyzl*3 was found to be expressed in testes and pancreas [16]. Sperm lysozyme-like protein 1 (SLLP1 or LYZL3) was found to be an intra-acrosomal and non-bacteriolytic c-type lysozyme-like protein in human spermatozoa [17,18]. LYZL4 is a sperm bound protein with a role in fertilization [19] and is expressed in testes and epididymis. Along with cystatin-c, cystatin 8 and premelanosome protein, presence of lysozyme-like 1, 3, 4 and 5 (LYZL1, LYZL3, LYZL4 and LYZL5) proteins in the acrosomal matrix of the mouse spermatozoa was reported [20]. The LYZL proteins that are similar to lysozyme also possess the amyloidogenic domains suggesting that they may play important role like CRES proteins. Alpha lactalbumin, otherwise called as LYZL7 belongs to glycoside hydrolase family and is a homologous to lysozyme. Recently, a modified form of lactalbumin HAMLET (human lactalbumin made lethal to tumors) was reported to have apoptotic activity against tumor cells [21]. *Lyzl3/Sllp1* was found to be expressed specifically in the male reproductive tract [18]. Spermatozoa incubated with antibodies to human SLLP1 failed to fertilize eggs, thereby demonstrating a role in male reproductive function. Microscopic studies revealed that LYZL3 is located in the acrosomal region before capacitation and moves towards the equatorial segment after capacitation, suggesting that LYZL3 may be an intra-acrosomal protein that is tightly bound to the sperm membrane. Further, interaction studies performed using LYZL3 and oocyte lysate revealed that it may interact with SAS1B, an oolemmal protein [22] and the same was confirmed by surface plasmon resonance. Similarly, incubation of spermatozoa with the mouse LYZL4 antibodies resulted in loss of fertilizing ability [19]. In the mouse, LYZL6 was reported to be present in testes, epididymis and spermatozoa and is antimicrobial in nature [23].

We previously reported the gene expression pattern of some rat *Lyzl* genes with emphasis on *Lyzl4* [24]. However in-depth analysis of their expression pattern and biochemical functions in general physiology and in the male reproductive function in particular are lacking. Hence, in this part of the study we attempted to characterize the expression of rat *Lyzl* genes and their protein products. Biochemical characterization was also undertaken to understand their role in general physiology.

## Materials and Methods

### *In silico* analyses

Using chicken lysozyme gene sequence, the rat genome (build RGSC v3.4) at NCBI was searched to identify the *Lyzl* genes. The sequences were then analyzed using *in silico* tools to predict various properties of the genes and their proteins (S1 Table). Genomic neighborhood analysis was performed based on the genome assemblies deposited in NCBI and Ensembl. Phylogenetic tree was constructed using neighborhood joining method to understand the evolution of rat LYZL proteins and their conservation among various organisms. Unrooted tree was constructed with the maximum sequence difference of 0.85. Domain / motif prediction was performed for rat LYZL proteins using Multiple Em for Motif Elicitation program (http://meme.nbcr.net/meme/cgi-bin/meme.cgi) [25]. The secondary structure of rat LYZL proteins was analyzed by Self-Optimized Prediction Method with Alignment (SOPMA) [26] to predict the possible secondary structures like α-helix, β-turn, extended strand and random coil. The program gives the output in the form of percentage of each secondary structure present in the given protein sequence [27].

### Molecular modeling

SWISS MODEL, accessible via the ExPASy web server, or from the program Deep View (Swiss Pdb-Viewer) was used to predict the three dimensional models of LYZL proteins. Full length sequence (without the signal peptide) of the LYZL proteins in FASTA/Raw format or SwissProt / UniProt accession code that are retrieved from NCBI were given as an input for the program. The output was generated as a 3D model using nearest homolog as a template with higher sequence identity using BLOSUM 45, 62, 80 at different Pfam value [28]. The models generated were validated using PROCHECK [29]. Proteins showing more than 90% in the core region and not more than 5% in the disallowed region are considered as good model. In cases where the models does not meet the requirements of quality structure, modeling was repeated after loop refinement and energy minimization and the structures were validated by analyzing the stereochemical features using PROCHECK [30]. PYMOL was used to visualize the structure of the modelled rat LYZL proteins.

### Docking analysis

GOLD (Genetic Optimization for Ligand Docking) program was used to analyze the binding ability of LYZL proteins to N-acetyl glucosamine (NAG) trisaccharide [31]. The flow chart of analysis is given in the S1 Fig. To the 3D models generated in this study, hydrogen molecules were added to the receptor and docking (rigid and flexible) performed with tautomers of the ligand and a particular atom number was given from the identified active site with a radius of 10.0 Å. All the other fitness function parameters and the genetic algorithm parameters were kept in default mode. The GOLD was run and the output was viewed using Goldmine and Silver. The output was produced as GOLD Fitness scores and different energy functions. The GOLD fitness scores of 40 and above generated using Goldmine and Silver were mainly considered. The output of these protein-ligand complexes were exported as PDB files using GoldMine. These complexes were then analyzed using molecular graphics viewers Discovery studio or PyMOL. The output was analyzed for the properties such as hydrogen bonding and Vander Waal’s interaction using LigPlot [32].

### Polymerase chain reaction

Wistar rats aged 90 days were obtained from National Institute of Nutrition, Hyderabad. 2 μg of total RNA isolated from brain, heart, lungs, liver, kidney, spleen, ovary, uterus, cervix, caput, corpus, cauda, testis, seminal vesicles and prostrate was reverse transcribed and using gene specific primers (S2 Table), the mRNA expression of rat *Lyzl5* and *Lyzl7* was analyzed using standard PCR conditions (94°C for 1 min followed by 30 cycles at 94°C for 30 sec, 58°C for 30 sec and 72°C for 30 sec, and with a final round of extension at 72°C for 10 min). The internal control gene glyceraldehyde 3 phosphate dehydrogenase (*Gapdh*) was amplified with the same conditions. PCR amplified gene products were analysed by electrophoresis on 2% agarose gels.

To study the androgen regulation of *Lyzl* transcripts, epididymides were obtained from sham operated, castrated and testosterone supplemented Wistar rats (n = 5 in each group). Testosterone supplementation was by a 20 mg dihydrotestosterone pellet implanted subcutaneously immediately after castration. The animals were sacrificed 14 days after castration. All procedures involving animals were conducted using the guidelines for the care and use of laboratory animals and this study was specifically approved by the Institutional Animal Ethics Committee of University of Hyderabad (UH/IAEC/2012/AUG/2). For studies on the developmental regulation of *Lyzl* genes, testes and epididymides were obtained from 10- to 60-day old Wistar rats.

### Recombinant protein production

The full length cDNA of rat *Lyzl 1*, *3*, *4*, *5*, *6* and *7* without the signal peptide were amplified and cloned into pQE80m vector. Plasmid containing one of the *Lyzl* coding region was transformed into *E*.*coli BL21* and the recombinant protein expression was induced with 1mM IPTG for 3 hr. Recombinant protein was purified using Ni-NTA agarose affinity purification system as per the manufacturer’s instructions (Qiagen, Valencia, CA, USA). The purified recombinant protein with a tag (MRGSHHHHHHGS) at the N-terminus was confirmed by Western blotting using anti-His tag antibody (Santa Cruz Biotechnology, Dallas, USA; sc-57598 RRID: AB_831408). The fractions that contained the protein of interest were dialyzed extensively at 4°C against 10 mM sodium phosphate buffer, pH 7.4.

### Generation of polyclonal antibodies

Antibodies to LYZL proteins were raised as described earlier [33]. New Zealand white rabbits aged four months were obtained from National Institute of Nutrition, Hyderabad and immunized intradermally with 600 μg of the recombinant protein mixed with equal volume of Freund’s complete adjuvant. Booster doses containing 600 μg protein was mixed in Freund’s incomplete adjuvant were administered on 3^rd^ and 28^th^ day. Antibody titer was checked on 35^th^ day and final bleeding was done on 42^nd^ day. Each antibody was checked for cross reactivity against each and every LYZL protein to ensure there is no cross reactivity within recombinant LYZL proteins. We observed no cross reactivity for all the antibodies (S2 Fig). The antibodies generated are registered at the Resource Identification Portal (http://antibodyregistry.org/) with the following RRID numbers: LYZL1 –AB_2616571; LYZL3 –AB_2616572; LYZL4 –AB_2616573; LYZL5 –AB_2616574; LYZL6 –AB_2616575; LYZL7 –AB_2616576.

### Immunoblotting

Testes, caput, cauda, seminal vesicles and prostate tissues were collected from 90 day old Wistar rats and 10% homogenates were prepared in RIPA buffer (25mM Tris-HCL, pH 7.6; 150 mM NaCl; 1% each of NP-40, sodium deoxycholate and sodium dodecyl sulfate) containing protease inhibitors. For sperm lysate preparation, 5 X 10^6^ spermatozoa were used. The homogenates were then centrifuged at 10,000 rpm for 10 min to remove the debris and concentration of the protein in the supernatant was quantified by Lowry’s method. 100 μg of the total protein was separated by electrophoresis on 15% SDS PAGE and transferred to nitrocellulose membrane at 25V for 16 h. The membrane was then blocked with 5% skim milk for 2h at room temperature followed by probing with primary antibody (immune serum) for 1h, subsequently washed with TBS (Tris-buffered saline) and TBS-T (Tris-buffered saline, 0.1% Tween-20). It was then incubated with Goat anti-Rabbit IgG-HRP antibody (Santa Cruz Biotechnology, Dallas, USA; sc-2054 RRID: AB_631748) followed by washes with TBS and TBS-T each for 10 min. At the end of washing, the membrane was developed using enhanced chemiluminescence (GE Healthcare, Buckinghamshire, UK) kit.

### Immunofluorescence

Wistar rats aged 90 days were dissected to collect the testes, spermatozoa and epididymides. Testes and epididymides were fixed by immersing in Bouin’s solution and 4% paraformaldehyde solution respectively. Serial sections (five microns) of testes and epididymides were preheated to 60°C for 5 min followed by washings with xylene, gradient alcohol (70–100%) and PBS for 10 min each. Following antigen retrieval (by heating in 10 mM citrate buffer, pH 6.5 for 12 min), permeabilization with PBS containing 1% triton X-100 (PBST) for 15 min and blocking with 10% goat serum for 45 min, the sections were incubated with immune serum and Goat anti-Rabbit IgG tagged with TRITC or FITC (Santa Cruz Biotechnology, Dallas, USA; sc-2091 RRID: AB_649008; sc-2012 RRID: AB_631744) for 1 h each with washing with PBS in between incubations. 4', 6-diamidino-2-phenylindole (DAPI; Sigma Aldrich, St. Louis, USA)) was used to stain the nucleus and images taken using confocal microscope. In the case of spermatozoa, smears on glass slides were prepared and air dried. They were then permeabilized with PBST, blocked with 10% goat serum and processed in a similar way as that of tissue sections.

### Circular Dichroism

Circular dichroism was performed for recombinant LYZL proteins using Jasco J-810 spectropolarimeter. Quartz cell with a path length of 0.2 cm was loaded with 200 μl of 0.1μg/μl protein and scanned in the far UV region (180–260 nm). Three scans at a scan speed of 50 nm/min were accumulated and the polarimetry data was collected for every 1 nm. The data thus collected was used for calculating mean residue ellipticity (MRE) and the spectrum was plotted [34]. In addition, the spectra of appropriate blank solution, 10 mM phosphate buffer was subtracted from the spectrum of the protein. Percentage of secondary structure elements were calculated using K2D3 tool of Dichroweb [35].

### Muramidase Assay

The muramidase assay is based on the cleavage of β-glycosidic bond between N-acetyl muramide and N-acetyl glucosamine [36]. Reduction in O.D at 450 nm is observed as the glycosidic bonds are broken. Recombinant rat LYZL protein was incubated with 2 ml of *M*. *lysodeikticus* cells (Sigma Aldrich, St. Louis, USA) in 50 mM KH_2_PO_4_-NaOH buffer, pH 7.0, and the decrease in turbidity was monitored at 450 nm in a spectrophotometer for every 60 min until 6 h. Change in O.D (Δ O.D) was calculated by subtracting the final O.D from initial O.D. Muramidase activity was expressed as Δ O.D. Lysozyme (Sigma Aldrich, St. Louis, USA) was used as a positive control.

### Isopeptidase Assay

The isopeptidase activity assay is based on the cleavage of L-γ-glutamine-p-nitroanilide (L-γ-Glu-pNA) to produce p-nitroanilide (pNA), which exhibits absorbance at 405 nm [37]. Recombinant rat LYZL proteins were added to the reaction mixture containing 1.75 mM L-γ-Glu-pNA (Sigma Aldrich, St. Louis, USA) in 0.05 M 3-(N-morpholino) propane sulfonic acid (MOPS) buffer, pH 7, containing 0.01M NaCl and the formation of pNA was monitored spectrophotometrically at 405 nm for every 1 h until 6 h. Activity was expressed as Δ O.D. Lysozyme was used as positive control.

### Antibacterial Assay

Colony forming units (CFU) assay was employed to test the antibacterial activity [38]. Briefly, overnight cultures of *E*. *coli* XL-1 blue were grown to mid-log phase (A_600_ = 0.4–0.5) and diluted with 10 mM sodium phosphate buffer (pH 7.4). Approximately 2 X 10^6^ CFU/ml of bacteria was incubated at 37°C with 10–100 μg/ml of the recombinant LYZL protein and aliquots of the assay mixture were taken at 30, 60, 90 and 120 min after the start of incubation. The assay mixtures were serially diluted with 10 mM sodium phosphate buffer (pH 7.4) and 100 μl of each was spread on a Luria–Bertani agar plate and incubated at 37°C overnight to allow full colony development. The resulting colonies were hand counted and plotted as log CFU/ml. Lysozyme was used as a positive control.

### Scanning Electron Microscopy

*E*.*coli* were treated with recombinant LYZL protein for 120 min and the bacterial cells were fixed in Karnovks’s fixative solution (2% paraformaldehyde, 2.5% glutraldehyde in 0.1M phosphate buffer) for overnight at 4°C. They were then serially washed with graded alcohol (30% to 100%) for dehydration and finally suspended in acetone before embedding on carbon tape. The samples were then coated with gold and then observed under scanning electron microscope [39]. Lysozyme was used as a positive control.

### Membrane potential measurement

The effect of LYZL protein on the bacterial membrane potential and permeability was determined by using DiOC_2_(3) and TO-PRO-3 respectively (Sigma Aldrich, St. Louis, USA) [40]. DiOC_2_(3) emits green fluorescence as a single molecule, which varies with cell size and is independent of membrane potential. The red fluorescence of DiOC_2_(3) is due to dye aggregation and depends on both size and membrane potential. Therefore the ratio of red to green is attained to measure the membrane potential. TO-PRO-3 is a DNA binding dye and is impermeable to live cells. Due to its ability to enter into membrane compromised cells it is used as dead cell indicator. The dead cells are eliminated when measuring the fluorescence of DiOC_2_(3). *E*. *coli* grown to mid log phase were diluted in 10 mM sodium phosphate buffer pH 7.4 to a final concentration of 10^6^ to 10^7^cells / ml and then treated with 100 μg of recombinant LYZL protein or 15 μM of the bacterial membrane potential disruptor carbonyl cyanide m-chlorophenyl hydrazine (CCCP; Sigma Aldrich, St. Louis, USA) for 2 h at 37°C in orbital shaker. The cells were then washed in 10 mM phosphate buffer, pH 7.4 followed by incubation with 30 μM DiOC_2_(3) and 100 nM TO-PRO-3 for 5 min at room temperature. At the end of incubation, bacterial cells were washed and analyzed in a flow cytometer (BD LSR Fortessa). The far red fluorescence of TO-PRO-3 is measured in the PerCP-Cy5-5A. The green and red fluorescence of dye DiOC_2_(3) was measured in FITC and PE-Texas Red-A channel respectively.

### Peptidoglycan binding assay

96 well plates coated with 40 μg/ml peptidoglycan (PGN; Sigma Aldrich, St. Louis, USA) or hyaluronan (Sigma Aldrich, St. Louis, USA) were incubated at 37°C and at 60°C for overnight and 30 min respectively. The plates were then blocked with 1 mg/ml BSA for 2 h and varying concentrations of the recombinant LYZL protein was added to the wells and incubated for 3 h. The wells were then washed 4 times with PBS-T (PBS with 0.1% Tween-20), followed by sequential incubation with primary antibody against the LYZL protein being tested and Goat anti-Rabbit IgG-HRP conjugated antibody (Santa Cruz Biotechnology, Dallas, USA; sc-2054 RRID: AB_631748). After thorough washing, O-Phenylenediamine (OPD; Sigma Aldrich, St. Louis, USA) was used to measure the amount of antibody bound to the protein complex and the binding efficiency is measured in terms of ELISA index (EI). ELISA index is calculated by dividing the average O.D of test samples with average O.D of control samples [41]. Lysozyme was used as a positive control.

### Hyaluronidase activity assay

0.8% hyaluronan in 300 mM phosphate buffer (pH 7.4) was mixed with melted agarose (0.8%). 100 μl of the gel was dispersed into each well of a microtitre plate. After solidification 50 μl phosphate buffer (pH 7.4) containing varying concentrations of recombinant LYZL protein was added to each well and incubated for 17 h. At the end of the incubation, solutions were removed and the hyaluronan was precipitated by adding 100 μl of 10% cetyl pyridinium chloride and incubating for 30 min at 37°C. The turbidity developed was read at 595 nm [42]. Hyaluronidase activity was measured in terms of decrease in turbidity (or O.D). Hyaluronidase was used as a positive control.

### Free radical scavenging assay

This assay is based on the ability of 1,1-diphenyl-2-picrylhydrazyl (DPPH; Sigma Aldrich, St. Louis, USA)) to undergo reduction due to the presence of an odd electron, and thus exhibits a strong absorption maximum at 517 nm [43]. As this electron becomes paired off in the presence of a hydrogen donor, a free radical scavenging antioxidant, the absorption capacity decreases, resulting in decolorization. 0.04% of 1, 1 diphenyl-2-picryl hydrazyl (DPPH) was dissolved in methanol. To 100 μl of DPPH solution, varying concentrations of recombinant LYZL protein was added and incubated at room temperature for 30 min. Free radical scavenging capacity was assessed by measuring the discoloration of DPPH at 517 nm. Lysozyme was used as a positive control.

### Statistical Analysis

Statistical analysis was performed using ANOVA and Student’s t-test available in Sigma Plot software (SPSS Inc., Chicago, IL, USA). Values shown are mean ± SD.

## Results

### *In silico* characterization

**Fig 1:**
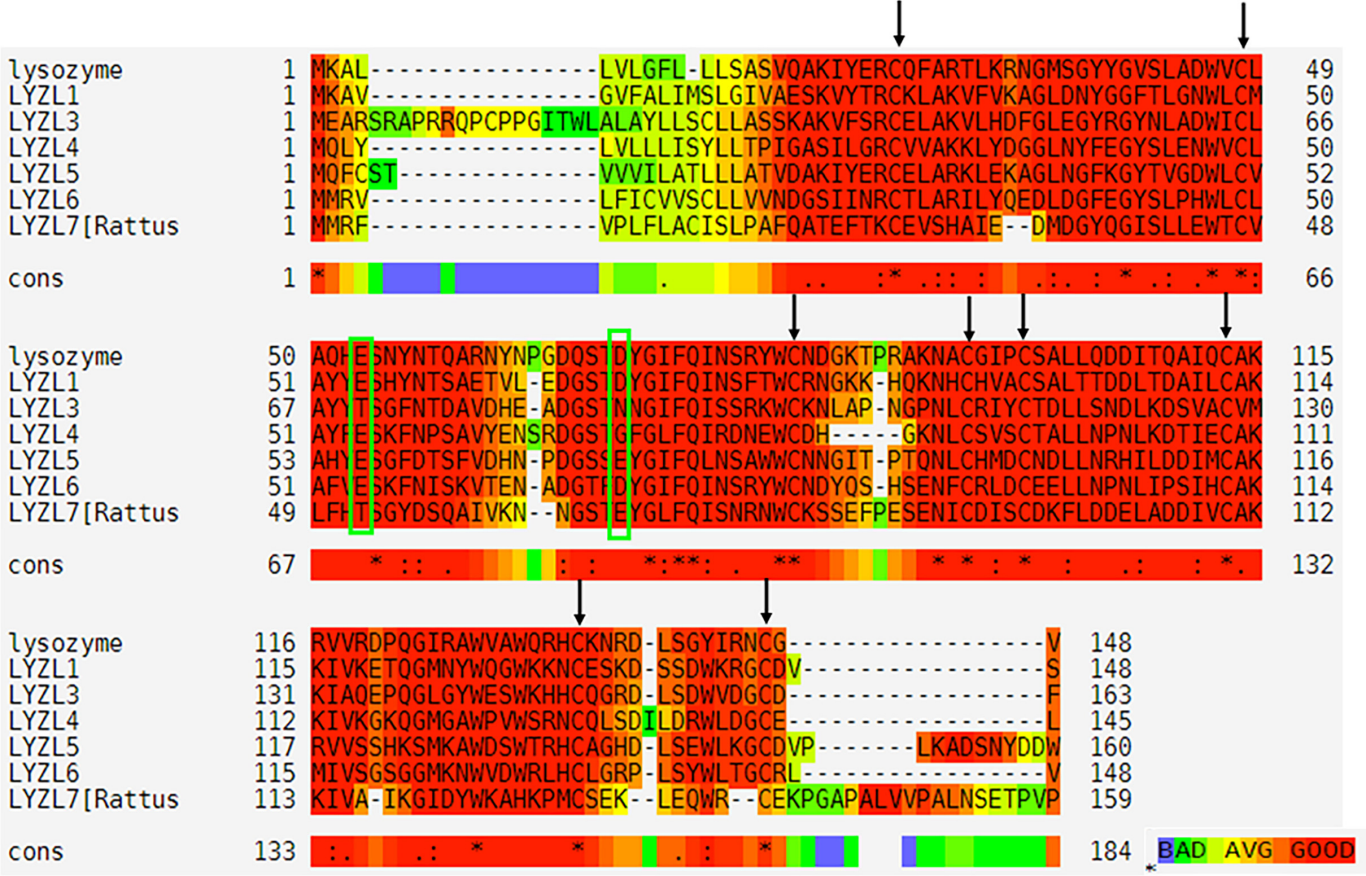
Alignment of rat lysozyme and LYZL proteins. Green color boxed amino acids represent the active site and black arrows indicate the conserved 8 cysteines. Sequence alignment of some rat LYZLs was reported by us earlier [24] and this figure presents a comprehensive analysis of all rat LYZLs.

We previously characterized the general features of some rat *Lyzl* genes and LYZL proteins [24]. In this study, the newly identified *Lyzl* genes and proteins along with those reported earlier were extensively characterized. *In silico* analysis of *Lyzl* genes were carried out and the attributes of the proteins were analyzed using different computational tools (S1 Table). *Lyzl* genes are located on different chromosomes and are not clustered, suggesting that they are not isoforms coded from a single gene. The LYZL proteins are hydrophilic, with molecular weight ranging between 16–18 kDa, with pI values between 4 to 8 (Table 1), possessed signal peptide and the conserved four disulfide bridges formed by 8 cysteine residues characteristic to lysozyme. LYZL1 and LYZL6 conserved the chicken lysozyme active site residues namely Glu54 and Asp71, whereas LYZL4 and 5 conserved only Glu54 and none were conserved in LYZL3 and LYZL7. Except for LYZL7, none of the LYZL proteins have calcium binding site though, they have the calcium binding domain. Glycosylation sites are present only in LYZL5 and LYZL7. Phosphorylation sites were found in all the LYZL proteins. Multiple sequence alignment using T-COFFEE program indicated high similarity of the amino acid sequence among the LYZL proteins (Fig 1). It is interesting to note that though there is high similarity, the amino acid sequences are not identical. CLUSTALW2 and BLAST program were used to determine the pairwise, similarity and comparison scores within the corresponding rat genes and the same are indicated in cyan, green and yellow colored boxes respectively in Table 2. Rat LYZL proteins seem to be more similar to their mouse counterparts than with the human counterparts (Table 2). Highest identity was exhibited by LYZL4 and LYZL6 in all the three species. The similarity score among the rat LYZL proteins was found to be in the range of 45–65 percent, whereas the identity lies between the 27–43 percent (Table 2).

**Table 1:** General predicted and deduced features of rat *Lyzl* genes and their encoded proteins^$^.

Attribute	*Lyzl1*	*Lyzl3*	*Lyzl4*	*Lyzl5*	*Lyzl6*	*Lyzl7*
**Chromosome**	17	10	8	X	10	7
**Gene accession number**	NC_005116.2	NC_005109	NC_005107.2	NW_047933.1	NC_005109	NC_005106.2
**Gene length**	13425	7560	4427	3184	5486	2699
**Total no. of exons**	5	6	4	4	4	4
**mRNA accession number**	NM_001108882.1	NM_001105820	XM_343507.4	NM_001108058.1	NM_001135833	NM_012594.1
**Length of mRNA**	855	939	580	553	801	480
**CDS**	143–589	333–824	1–438	1–483	81–527	1–480
**Protein accession number**	NP_001102352.1	NP_001099290.1	XP_343508.4	NP_001101528.1	NP_001129305.1	NP_036726.1
**Number of amino acids**	148	163	145	160	148	160
**Molecular Weight (KDa)**	16.5	18	16.3	18.1	17	17.8
**Localization**	secretory	secretory	secretory	secretory	secretory	secretory
**Isoelectric point (pI)**	8.38	6.4	5.79	5.36	5.74	4.74
O-glycosylation sites*	Nil	Nil	Nil	90,92	Nil	156
**GRAVY index**	-0.305	-0.299	-0.002	-0.241	0.117	-0.117
**Phosphorylation sites** *	19,55,135,138,59, 102,53,57,72	32,39	70,133,63	S77,82,140,141, 144, 150,175, T-8,81,Y-177	88,81,86	S-53,82,T-23,Y-37,55
**Signal peptide** *	1 to 19	1 to 36	1 to 19	1 to 21	1 to 19	1 to 19
Disulfide bonds*	25–145,49–133, 83–98,94–112	41–161,65–149, 99–114,110–128	25–143,49–30, 84–95, 91–109	27–147,51–135, 85–100,96–114	25–145,49–133, 83–98,94–112	25–139, 47–130,80–96,92–110
Active site*	Conserved (54,71)	Not conserved	Partially conserved (54)	Partially conserved (54)	Conserved (54,71)	Not conserved
Calcium binding site*	Nil	Nil	Nil	Nil	Nil	97,108

* -numbers indicate the position(s) of amino acid(s) in the protein.

^$^—The general features of LYZL proteins were reported by us earlier [24] and this table includes the previously reported data and additional analyses.

**Table 2:** Pairwise identity and similarity analysis of rat, mouse and human LYZL proteins.

	RAT						MOUSE						HUMAN					
	LYZL1	LYZL3	LYZL4	LYZL5	LYZL6	LYZL7	LYZL1	LYZL3	LYZL4	LYZL5	LYZL6	LYZL7	LYZL1	LYZL3	LYZL4	LYZL5	LYZL6	LYZL7
LYZL1	100	65	59	66	55	55	81	69	61	59	60	58	76	61	58	63	56	60
LYZL3	42	100	60	65	59	59	40	89	64	64	62	62	40	73	59	64	62	60
LYZL4	43	43	100	64	47	49	41	46	91	62	60	53	40	43	73	61	59	46
LYZL5	41	38	41	100	62	58	39	39	39	87	64	60	40	41	36	78	66	58
LYZL6	37	37	47	42	100	55	40	43	47	43	82	54	39	43	43	42	71	56
LYZL7	26	27	30	27	23	100	32	32	34	28	27	70	32	32	30	31	27	67

**All scores expressed in terms of percent.**

Cyan, orange and green color indicates **pairwise identity score, comparison with corresponding rat genes and similarity score respectively.**

**Fig 2:**
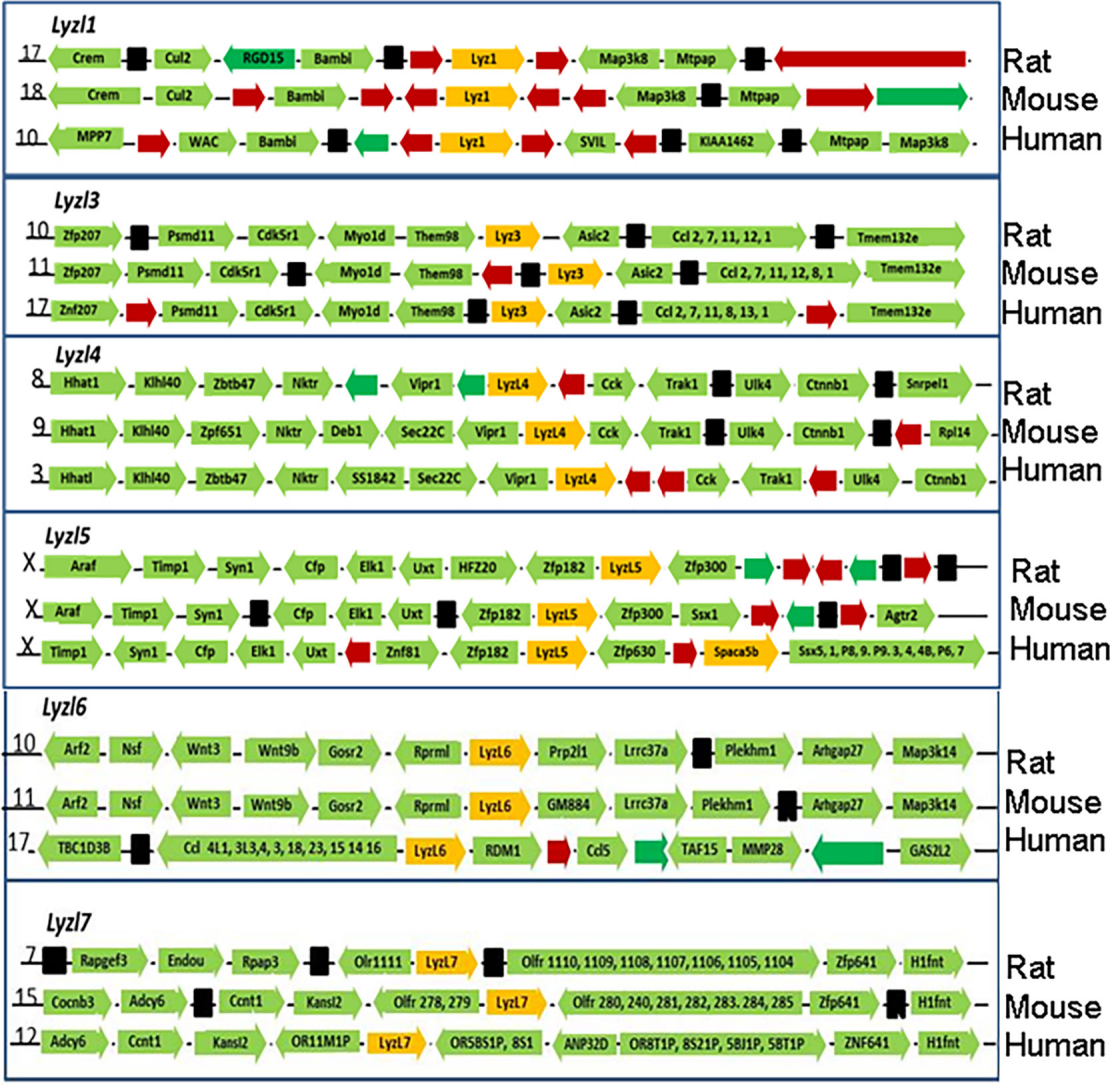
Genomic neighbourhood analysis of rat, mouse and human *Lyzl* genes. Green arrows with names represent the genes and directions of the arrow represent the direction of sense strand. Black, red and green arrows indicate noncoding, pseudo and hypothetical genes respectively. Numbers in the beginning of each row indicates the chromosome number and are shown in the order of rat, mouse and human.

Genomic neighborhood analysis revealed that the rat *Lyzl* genes in general are positioned among other genes similar to that of their mouse and human counterparts except for *Lyzl5* and *Lyzl6* (Fig 2). Neighborhood genes downstream of human *Lyzl5* gene are different from mouse and rat. A gene duplication event may have occurred due to which *Spaca5b*/*Lyzl5b* gene is present in case of human, but not in rat and mouse (Fig 2). Genomic neighborhood of *Lyzl6* for rat and mouse is almost similar. However, in humans, this gene is located in an entirely different neighborhood, suggesting that recombination may have taken place in the recent past after the evolution of mouse.

### Phylogenetic analysis of LYZL proteins

The presence of multiple *Lyzl* genes in all mammalian genomes as well as in the genomes of several other vertebrate species raises the possibility that the *Lyzl* gene family may have amplified early in vertebrate evolution. The phylogenetic analysis reveals that the LYZL proteins are present widely among various organisms especially the vertebrates (S2–S8 Figs). The rat LYZL proteins seem to have orthologs in rodents, placentals and primates.

### Secondary structure prediction

**Fig 3:**
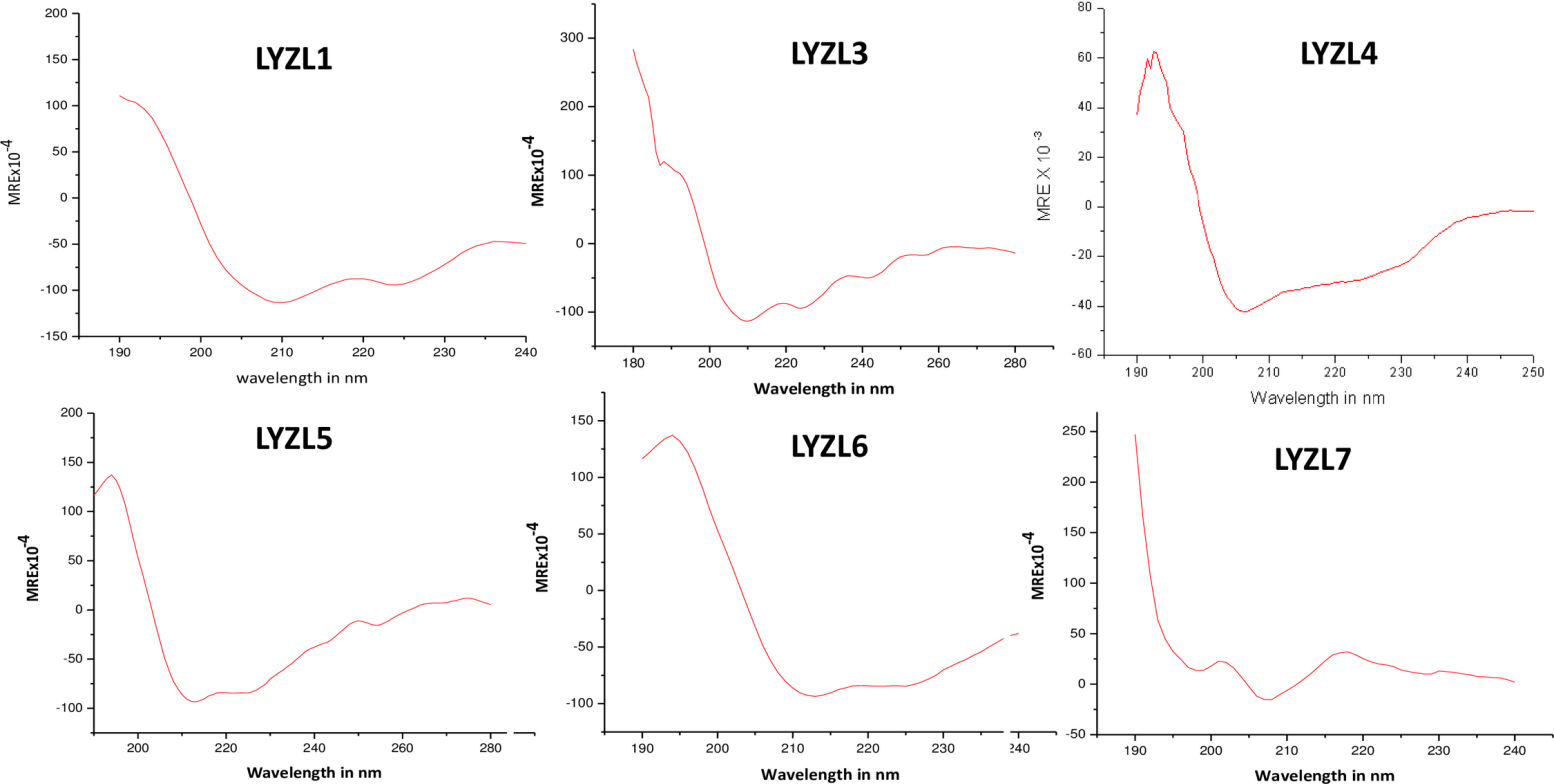
Circular dichroism spectra of recombinant LYZL proteins measured in terms of mean residue ellipticity (MRE).

The secondary structure prediction for rat LYZL proteins using SOPMA showed that LYZL 1, 5, 6 and 7 contains 42, 44, 44 and 44 percent of α-helix respectively, suggesting that they have predominantly α-helical pattern (Table 3). LYZL3 and 4 contains 39 and 55 percent of random coil and at the same time α-helix content is also comparably higher. Circular dichroism was performed to understand the folding of the recombinant LYZL proteins. The CD spectra of recombinant LYZL proteins (Fig 3) show a peak at 210 nm which is characteristic of α-helical protein. Further, mean residue ellipticity (MRE) values for each recombinant protein when tested using K2D3 (secondary structure analysis program) showed that these proteins contain α-helix pattern in their structure (Table 3).

**Table 3:** Secondary structure analyses by SOPMA and Circular Dichroism.

	SOPMA			Circular Dichroism			
Protein	α-helix	β-sheet	Coil	α-helix	Extended strand	β-sheet	Coil
**LYZL1**	87.41	0.21	12.38	41.89	15.54	7.43	35.14
**LYZL3**	43.1	1.9	45.3	36.81	15.3	8.59	39.26
**LYZL4**	54.6	2.63	42.77	37.9	21	14	55
**LYZL5**	60.95	0.19	38.86	44.38	16.88	8.12	30.63
**LYZL6**	65.89	1.05	33.06	43.92	18.24	9.46	28.38
**LYZL7**	23.39	24.55	52.06	42.77	15.09	5.66	36.48

### Molecular modelling

**Fig 4:**
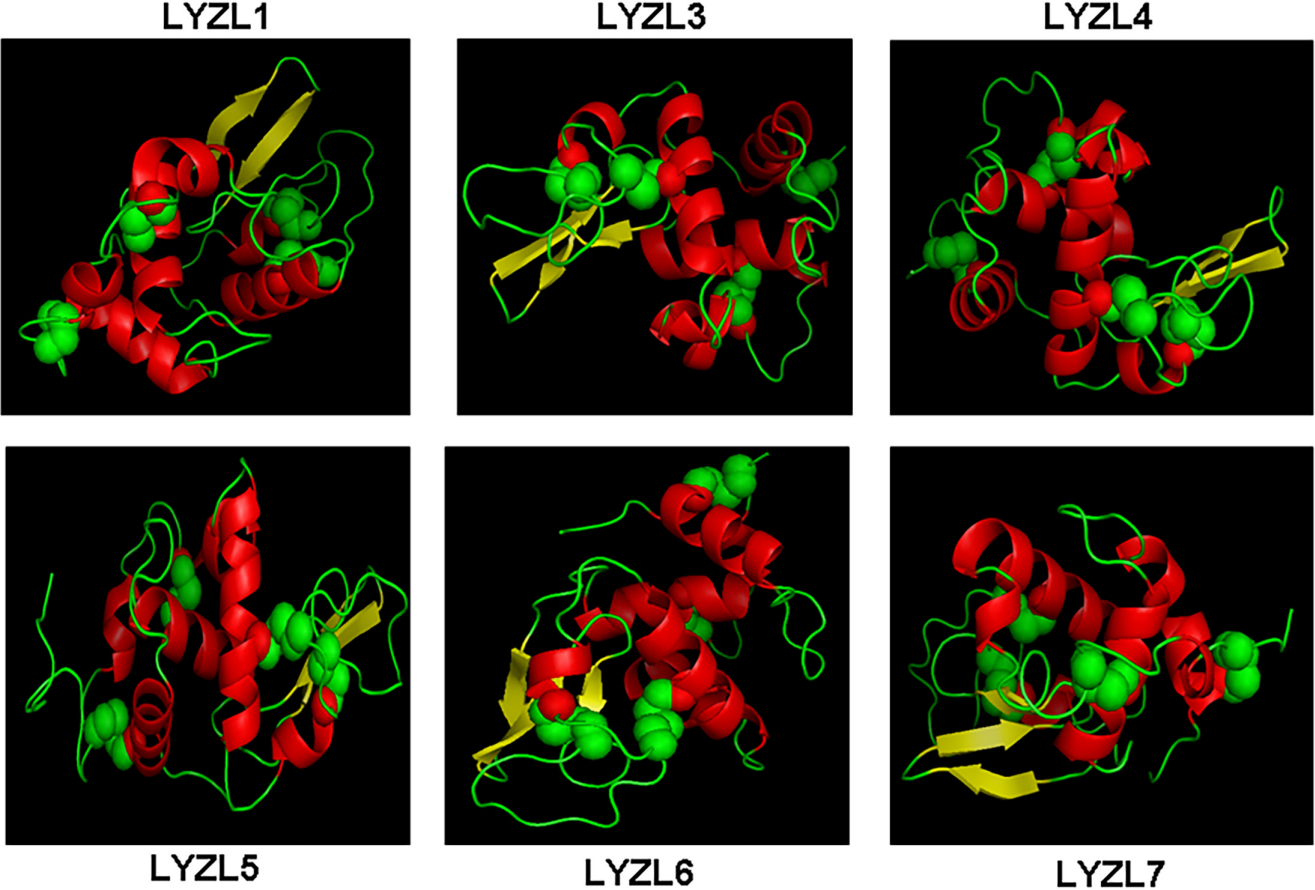
Modelled structure of LYZL proteins showing β- sheets in yellow, helices in red, loop regions in green and disulphide bonds in spheres.

The three dimensional structures (Fig 4) of LYZL proteins were predicted by SWISS MODEL and validated by PROCHECK. The reliability and correlation of the predicted model was measured in terms of G- factor and root mean square deviation (RMSD) between the template and the modeled protein. The templates used for generating these models, homology between the LYZL protein and the corresponding template, G-factor and RMSD values of the predicted 3D structures are detailed in Table 4. Ramachandran plot analyses indicate that majority of the amino acids of all the LYZL proteins are in the allowed and generously allowed regions. Further, the G-factor and RMSD values of all the LYZL proteins are in the acceptable limits.

**Table 4:** Templates used, G-factor, RMSD value and Ramachandran plot analyses for the 3D structures of rat lysozyme-like proteins.

	LYZL1		LYZL3		LYZL4		LYZL5		LYZL6		LYZL7	
Template used for generating 3D model; and the percent homology	Hen egg white lysozyme fused with fibrinogen; 58%		Mouse sperm lysozyme like protein1 (SLLP1); 86.61		Mouse sperm lysozyme like protein1 (SLLP1); 45%		Canine lysozyme; 48%		Mouse sperm lysozyme like protein1 (SLLP1); 45.67%		α-lacatalbumin; 86.7%	
G-factor / RMSD value	0.11 / 0.261		0.03 / 0.090		0.12 / 0.405		0.12 / 0.306		0.040 / 0.110		0.13 / 0.13	
	N	%	N	%	N	%	N	%	N	%	N	%
Most favoured regions [A, B, L]	83	71.6	98	88.3	99	90.8	96	82.4	97	86.6	94	83.2
Additional allowed regions [a, b, l, p]	31	26.7	13	11.7	10	9.2	17	14.4	15	13.4	18	15.9
Generously allowed regions [~a, ~b, ~l, ~p]	2	1.7	0	0	0	0	1	0.9	0	0	1	0.9
Disallowed regions [XX]	0	0	0	0	0	0	0	0	0	0	0	0
Non glycine and non proline residues	116	100	111	100	109	100	114	100	112	100	113	100
End residues (Excluding Gly and Pro)	2		2		2		2		2		2	
Glycine residues	10		11		12		10		9		6	
Proline residues	0		3		3		2		4		3	
Total number of residues	128		127		126		128		127		124	

N—indicates the number of residues in the protein.

### Molecular docking

**Fig 5:**
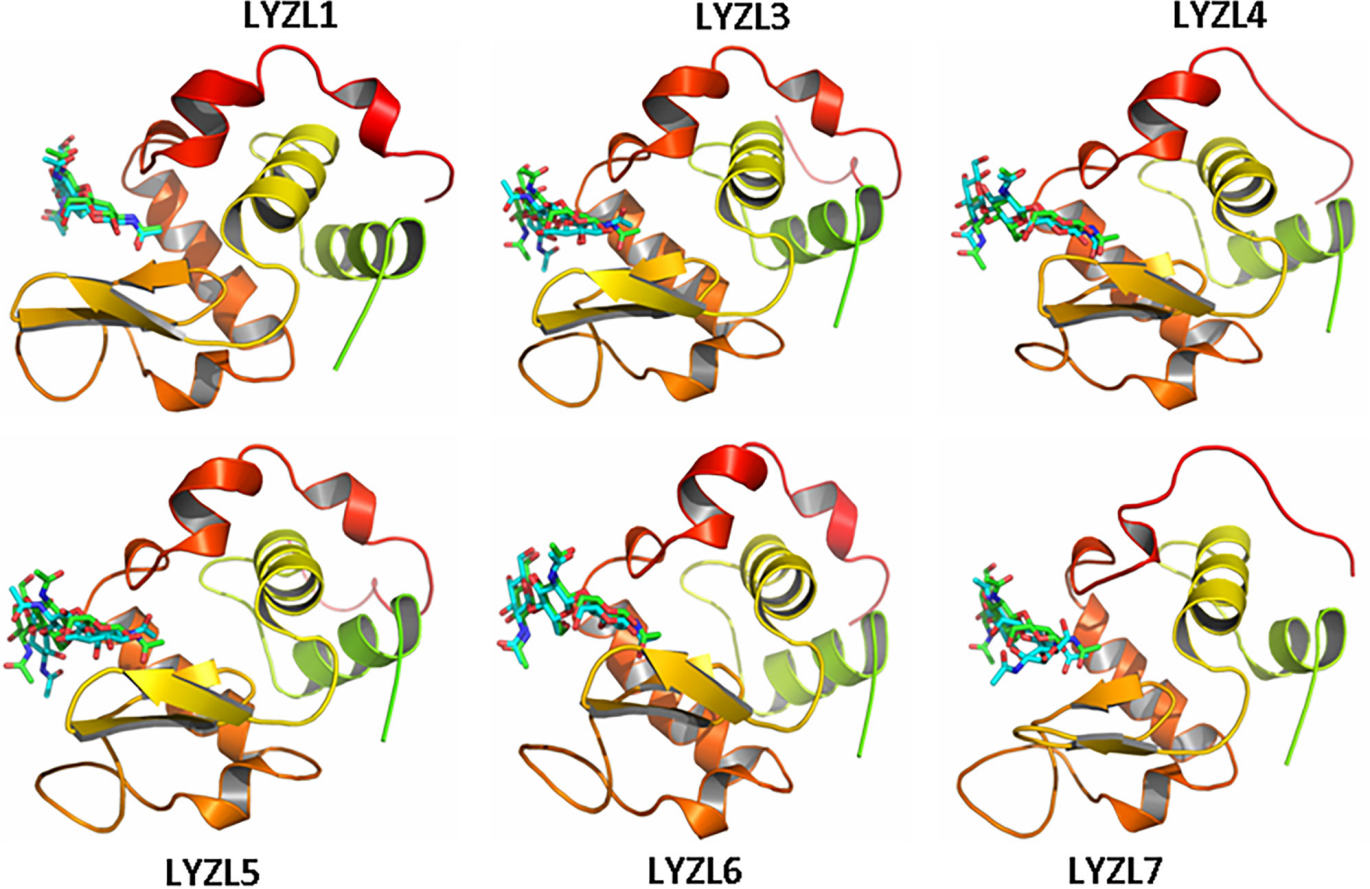
Molecular modeling showing the superimposition of rat LYZL and NAG complex with chicken lysozyme-NAG complex. NAG is represented in the form of stick model and the protein in cartoon model.

To gain further insights into the functional aspects of the LYZL proteins, docking of the LYZL proteins with N-acetyl-D-glucosamine (NAG), was carried out with chicken egg lysozyme bound to NAG as the template. Chicken egg lysozyme interacted with 11 amino acids which were primarily side and main chain interactions. The nature and number of amino acids that interacted with NAG (indicated with an underscore) varied among the LYZL proteins (Table 5). The remaining amino acids that were found to be interacting with NAG in lysozyme are replaced by similar but are not identical amino acids in the LYZL proteins. Further analyses were carried out to obtain docking score by GOLD. LYZL1 and LYZL6 had a score of 50.81 and 45.89 respectively. Such a high score indicates their ability to strongly interact with NAG. LYZL3, 4, 5 and 7 had docking scores of 26.58, 39.40, 18.92 and 22.9 respectively indicating weak interaction and this could be due to absence of the active site amino acids. LYZL proteins docked with NAG were superimposed with the chicken lysozyme bound to NAG trisaccharide. As shown in Fig 5, LYZL1 and LYZL6 displayed a complete superimposition with chicken lysozyme without any distortion of either the protein or ligand at any location. The remaining rat LYZL proteins did not show a complete overlap in the ligand region although there is complete overlap in the protein part indicating that these proteins may not interact with the substrate.

**Table 5:** Substrate interacting amino acids of chicken lysozyme and rat lysozyme like proteins.

Protein	Interacting amino acids and their positions										
**LYZ**	D	W	W	D	N	A	Q	D	I	L	W
**LYZL1**	D	T	W	E	Q	Y	Q	N	I	H	W
**LYZL3**	D	Y	W	G	G	N	Q	N	I	F	W
**LYZL4**	G	E	W	G	Q	A	Q	R	I	L	W
**LYZL5**	E	W	W	S	K	A	Q	N	L	L	W
**LYZL6**	N	K	W	E	Q	Y	Q	S	I	L	W
**LYZL7**	E	N	W	-	K	Y	Q	S	I	I	W

Underline indicates the substrate interacting amino acid that is similar to chicken lysozyme.

### *Lyzl* gene expression

**Fig 6:**
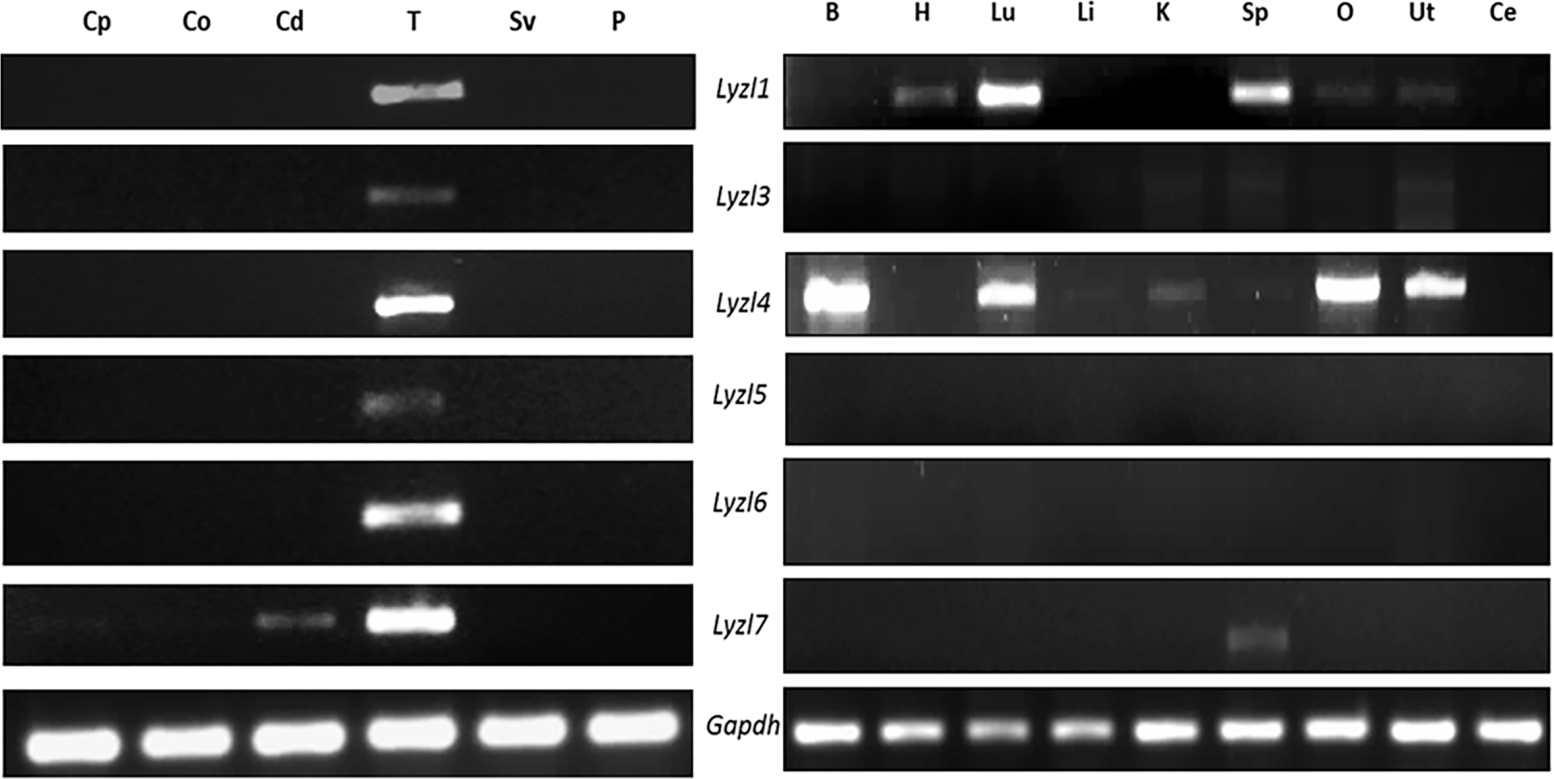
Expression of *Lyzl* genes in the male reproductive and non- reproductive tissues. RNA isolated from different tissues of rats were reverse transcribed and used for gene specific PCR. *Gapdh* was used as internal control. Cp-caput, Co-corpus, Cd-cauda, T- testes, Sv-seminal vesicle, P-prostate, B-Brain, H- Heart, Lu-Lungs, Li-Liver, K-Kidney, Sp-Spleen, O-Ovary, Ut-Uterus, Ce-Cervix, *Gapdh*-Glyceraldehyde 3 phosphate. Expression pattern of some rat LYZL genes were reported by us earlier [24]and this figure includes our previously reported data to present a comprehensive analysis of all rat LYZL genes.

**Fig 7:**
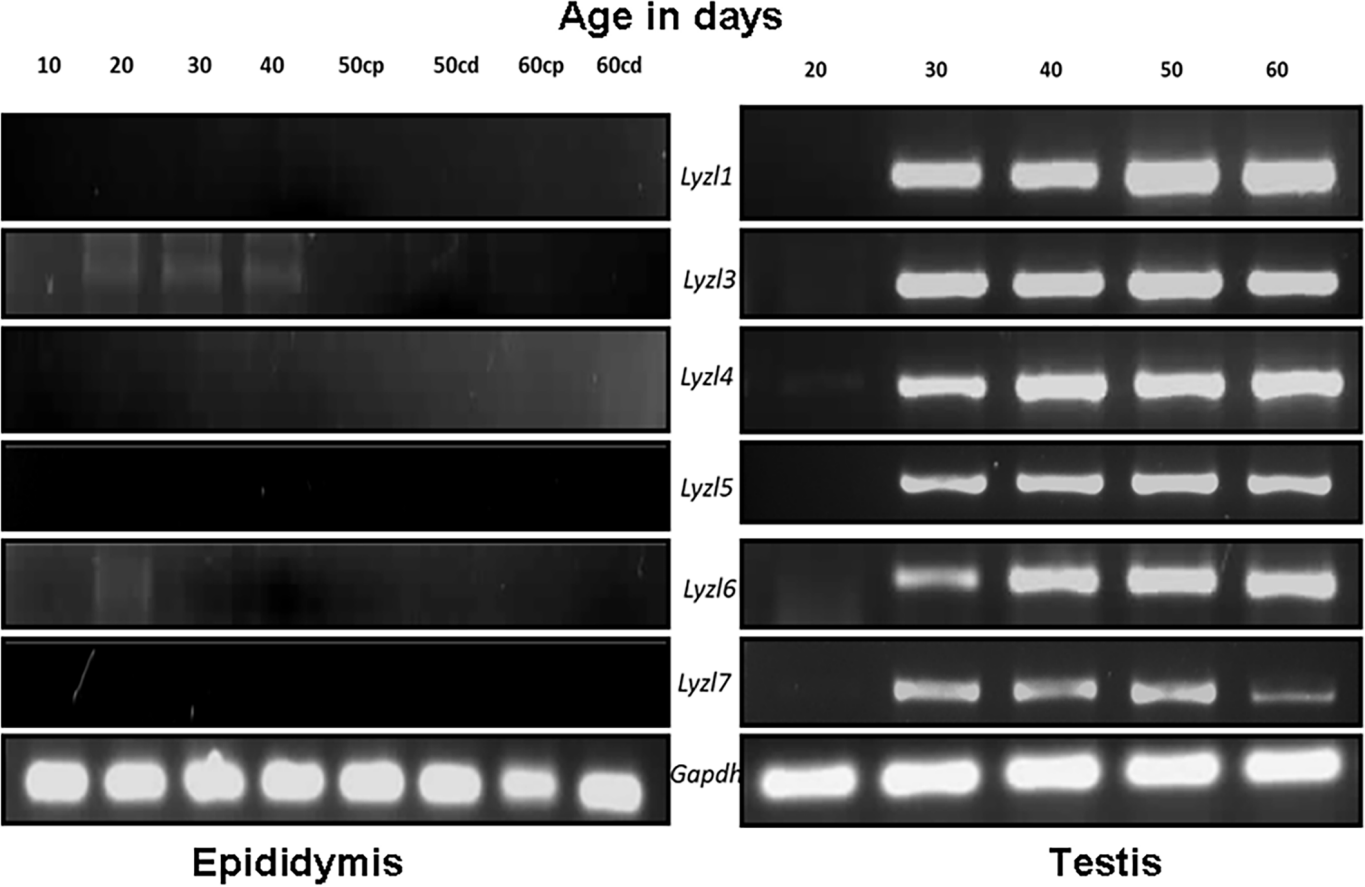
Developmental regulation of *Lyzl* genes in epididymidis and testes. RT PCR for *Lyzl* genes in epididymis and testes collected from rats of different age groups. 10, 20, 30, 40, 50 and 60 are the age of rats in days. cp- caput, cd-cauda. *Gapdh* was used as the internal control. Expression pattern of some rat LYZL genes were reported by us earlier [24] and this figure includes our previous data to present a comprehensive analysis of all rat LYZL genes.

In a previous study, we analyzed the mRNA expression pattern of *Lyzl1*, *Lyzl3*, *Lyzl4* and *Lyzl6* [24]. In this study, we report the mRNA expression pattern of *Lyzl5* and *Lyzl7* using gene specific primers in various rat tissues. We present the data of all *Lyzl* genes for better understanding. Among the male reproductive tract tissues, *Lyzl* genes were expressed only in the testis (Fig 6). However, *Lyzl7* expression was detected in the cauda along with testis. Further, PCR analysis using cDNA obtained from non-reproductive tissues and female reproductive tissues indicated that *Lyzl* 3, 5 and 6 transcripts are confined only to the testis. *Lyzl1*, *4* and *7* were found to be expressed in non-reproductive tissues as well (Fig 6). In the epididymis, *Lyzl* genes analyzed in this study were not detected at all the ages during development (Fig 7). In the testes, the *Lyzl* transcripts are expressed at all the age groups starting from 30 days (Fig 7).

### Expression of LYZL proteins

**Fig 8:**
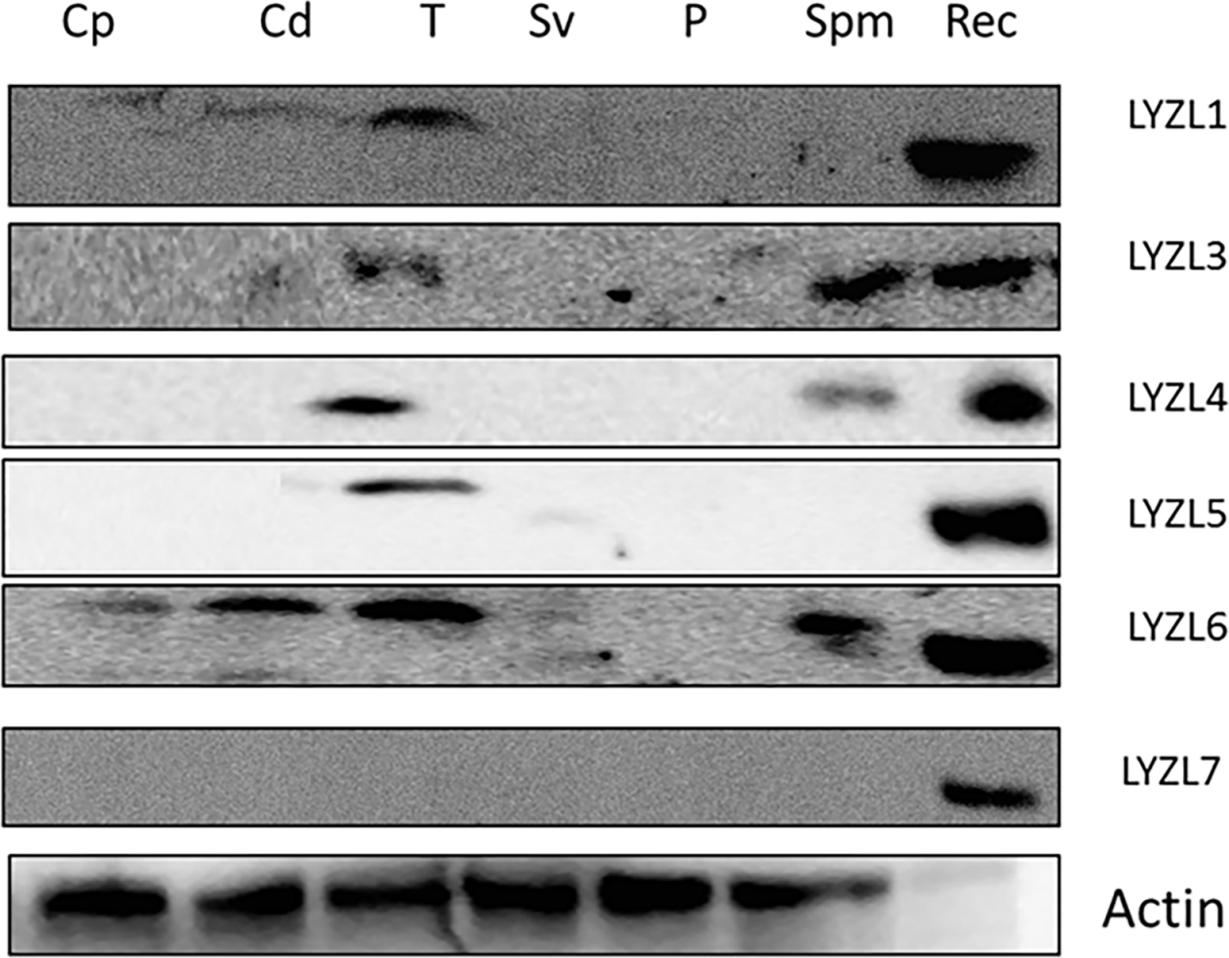
LYZL protein expression in male reproductive tract. Immunoblotting of LYZL proteins in male reproductive tract tissues. The immunoblots were probed with immune serum specific to each protein. Cp-caput, Cd-cauda, T-testes, Sv-seminal vesicles, P-prostate, Spm-sperm, Rec-recombinant protein.

Since mRNA expression of the *Lyzl* genes was found in the rat male reproductive tissues, their translation products (proteins) were also analyzed by immunoblotting. LYZL1, 3, 4 and 5 are expressed only in testes (Fig 8). LYZL6 is observed in both epididymis and testes. Only LYZL3, 4 and 6 were detected on sperm. LYZL7 expression was not detected in any of the tissues analyzed (Fig 8).

### Immunolocalization of LYZL proteins

**Fig 9:**
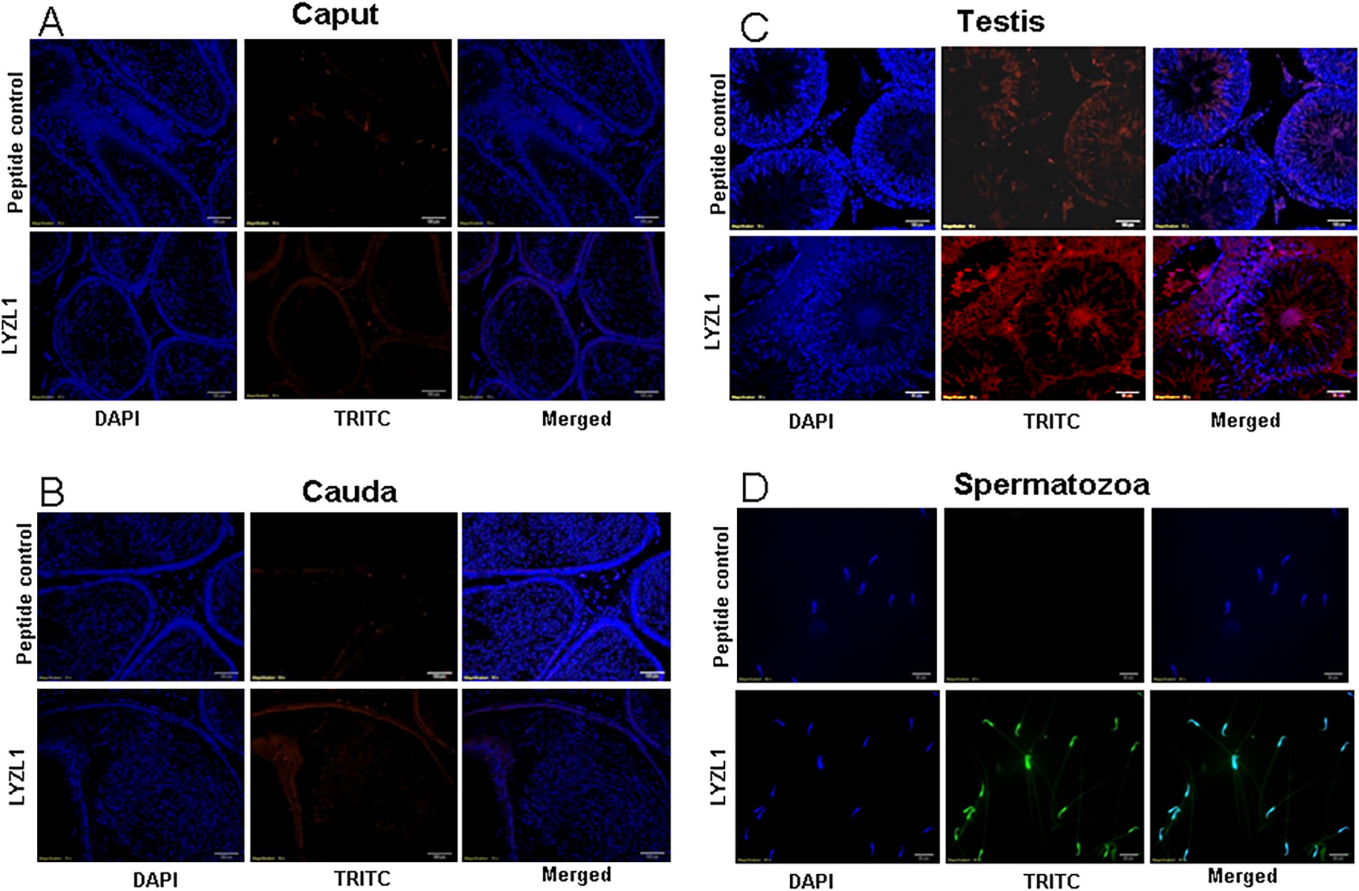
Immunolocalization of LYZL1. Serial sections of the rat testes and epididymides were incubated with antigen preadsorbed immune serum (peptide control) or immune serum raised against LYZL1, followed by TRITC (Tetramethylrhodamine-5-(and-6)-Isothiocyanate (5(6)) tagged secondary antibody and counter stained with DAPI (4',6-diamidino-2-phenylindole) nuclear stain. Spermatozoa were stained with FITC (fluorescein Isothiocyanate) tagged secondary antibody.

**Fig 10:**
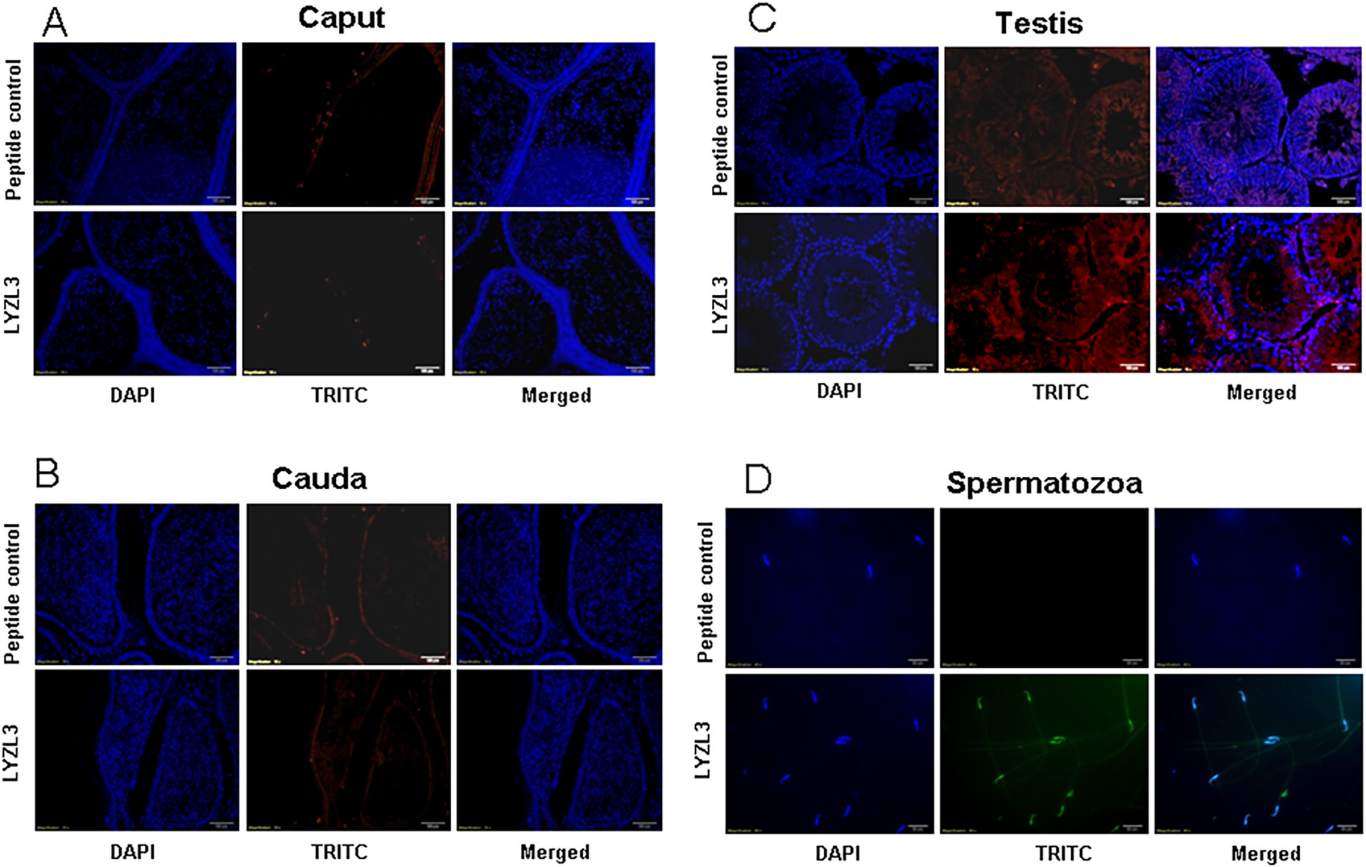
Immunolocalization of LYZL3. Serial sections of the rat testes and epididymides were incubated with antigen preadsorbed immune serum (peptide control) or immune serum raised against LYZL3, followed by TRITC (Tetramethylrhodamine-5-(and-6)-Isothiocyanate (5(6)) tagged secondary antibody and counter stained with DAPI (4',6-diamidino-2-phenylindole) nuclear stain. Spermatozoa were stained with FITC (fluorescein Isothiocyanate) tagged secondary antibody.

**Fig 11:**
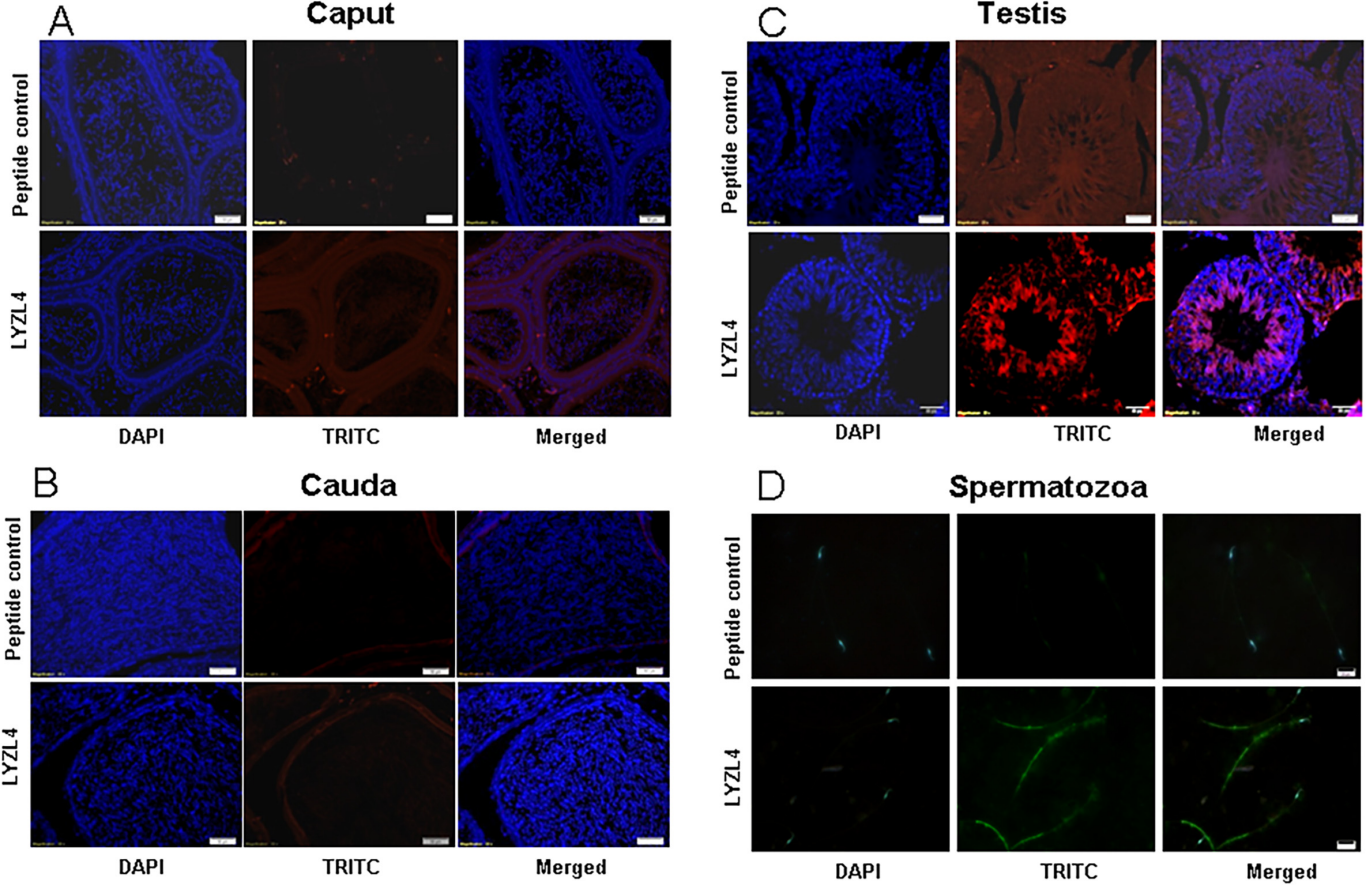
Immunolocalization of LYZL4. Serial sections of the rat testes and epididymides were incubated with antigen preadsorbed immune serum (peptide control) or immune serum raised against LYZL4, followed by TRITC (Tetramethylrhodamine-5-(and-6)-Isothiocyanate (5(6)) tagged secondary antibody and counter stained with DAPI (4',6-diamidino-2-phenylindole) nuclear stain. Spermatozoa were stained with FITC (fluorescein Isothiocyanate) tagged secondary antibody. Panel D showing LYZL4 localization was reported by us earlier [24] and the same figure is being used for a comprehensive presentation of results.

**Fig 12:**
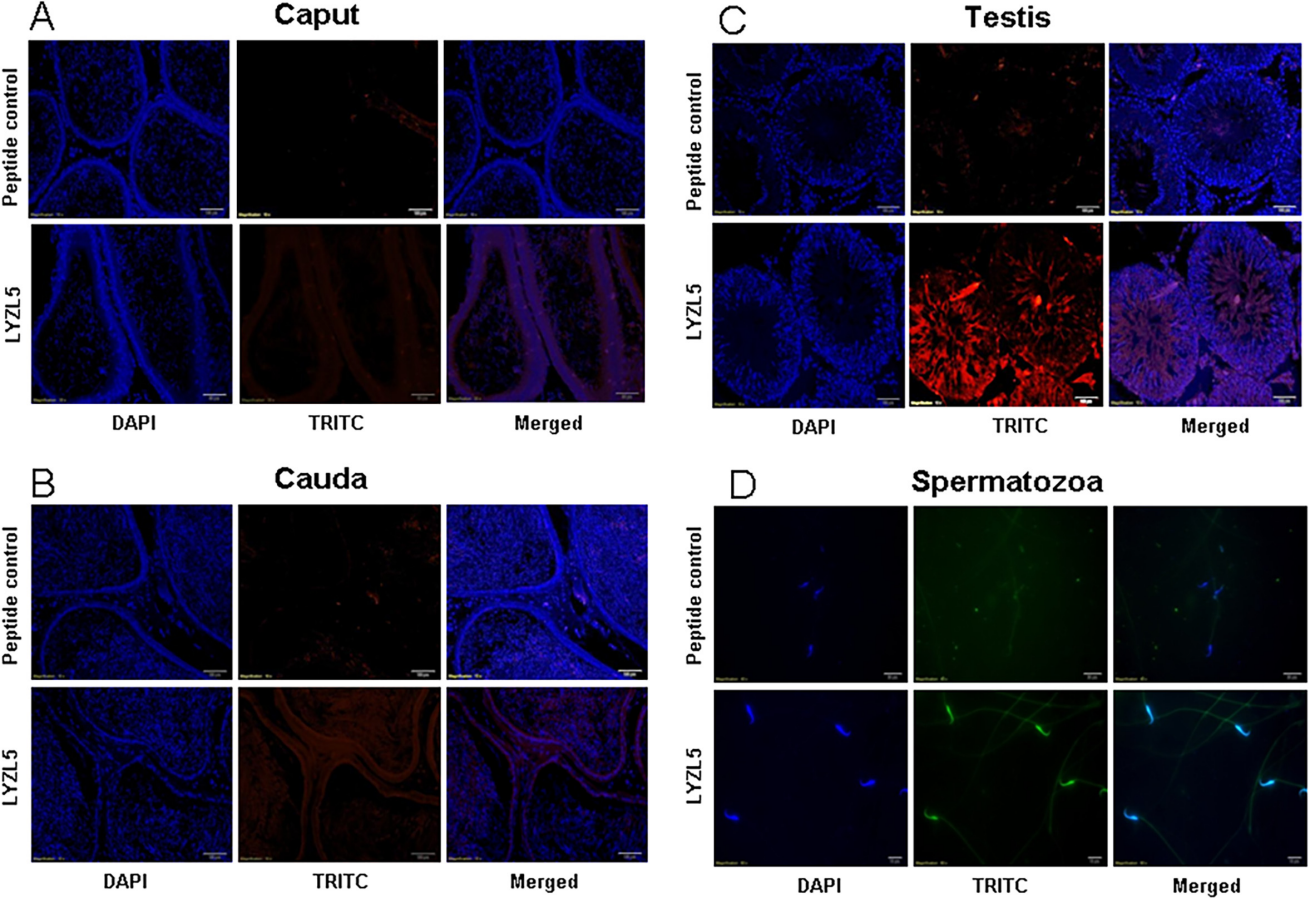
Immunolocalization of LYZL5. Serial sections of the rat testes and epididymides were incubated with antigen preadsorbed immune serum (peptide control) or immune serum raised against LYZL5, followed by TRITC (Tetramethylrhodamine-5-(and-6)-Isothiocyanate (5(6)) tagged secondary antibody and counter stained with DAPI (4',6-diamidino-2-phenylindole) nuclear stain. Spermatozoa were stained with FITC (fluorescein Isothiocyanate) tagged secondary antibody.

**Fig 13:**
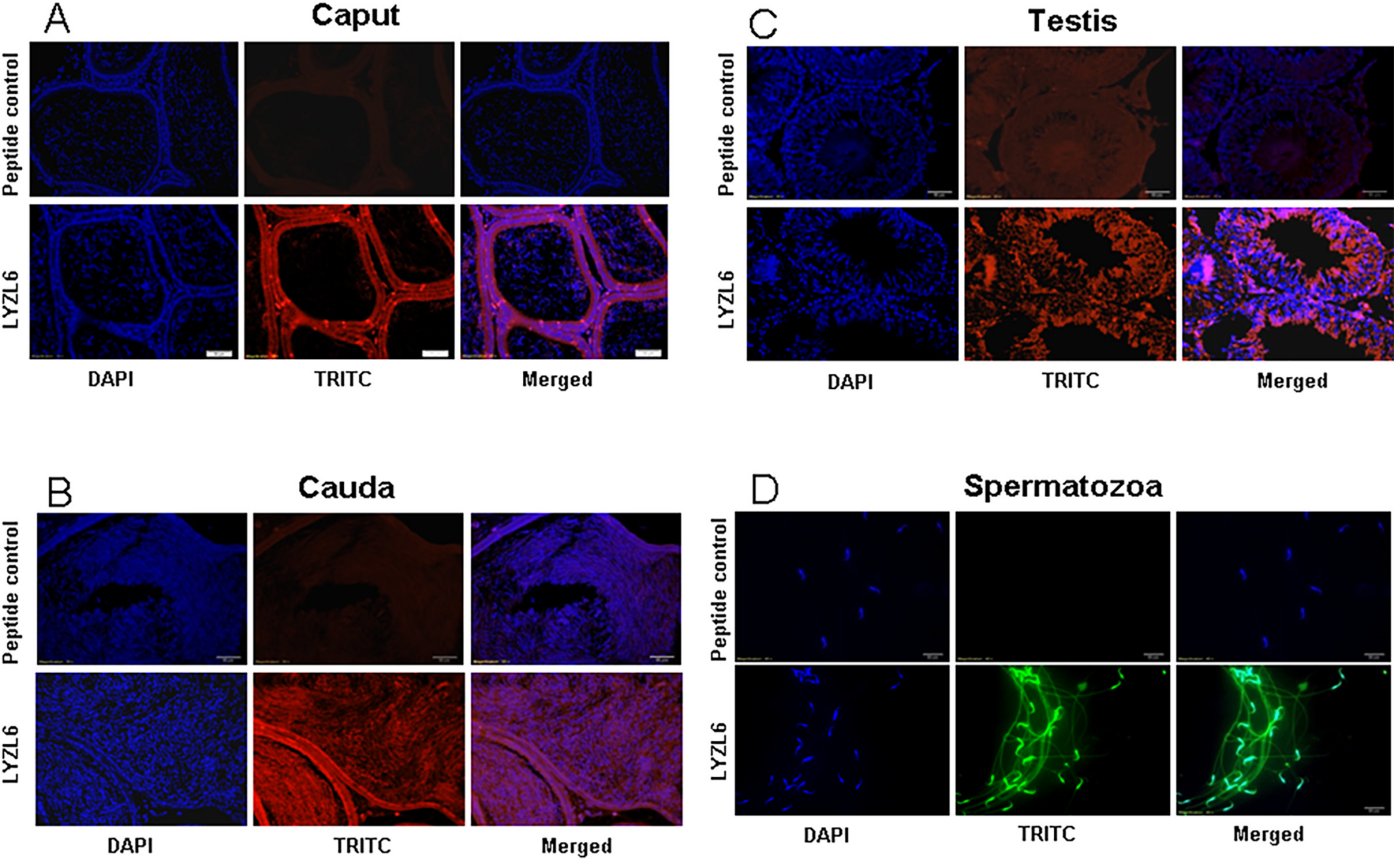
Immunolocalization of LYZL6. Serial sections of the rat testes and epididymides were incubated with antigen preadsorbed immune serum (peptide control) or immune serum raised against LYZL6, followed by TRITC (Tetramethylrhodamine-5-(and-6)-Isothiocyanate (5(6)) tagged secondary antibody and counter stained with DAPI (4',6-diamidino-2-phenylindole) nuclear stain. Spermatozoa were stained with FITC (fluorescein Isothiocyanate) tagged secondary antibody.

**Fig 14:**
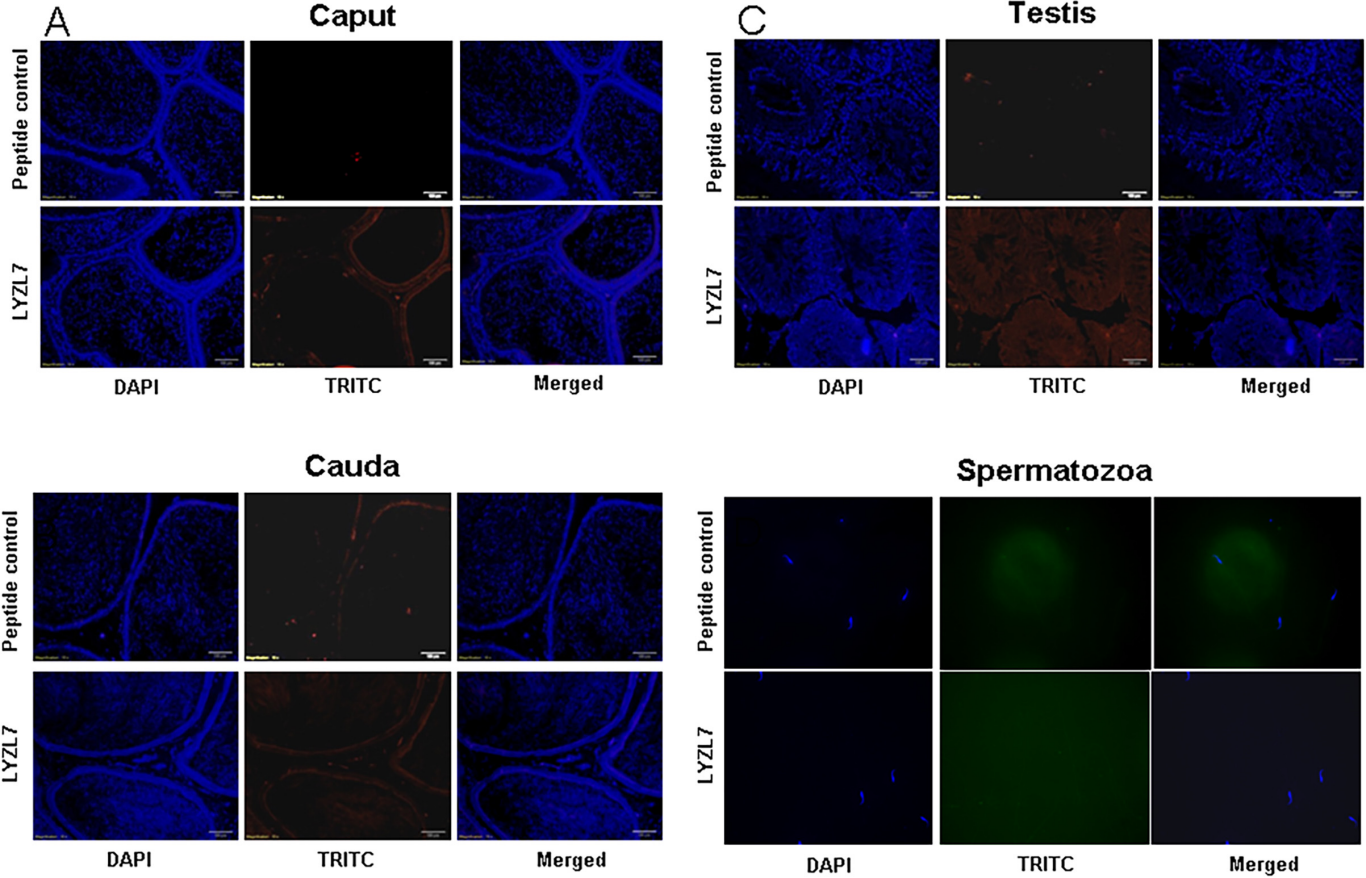
Immunolocalization of LYZL7. Serial sections of the rat testes and epididymides were incubated with antigen preadsorbed immune serum (peptide control) or immune serum raised against LYZL7, followed by TRITC (Tetramethylrhodamine-5-(and-6)-Isothiocyanate (5(6)) tagged secondary antibody and counter stained with DAPI (4',6-diamidino-2-phenylindole) nuclear stain. Spermatozoa were stained with FITC (fluorescein Isothiocyanate) tagged secondary antibody.

LYZL1 protein was localized only in the testes, especially in the germinal epithelium. It was also detected in the head region of spermatozoa obtained from adult rats (Fig 9). Similarly, LYZL3, 4 and 5 (Figs 10, 11 and 12) were found to be localized in the testes and on the spermatozoa. LYZL3 and 5 are localized to head region of the spermatozoa, whereas LYZL4 expression was restricted to tail region (Fig 11). The expression of LYZL4 on the spermatozoa was previously reported by us [24] and the same is being included in Fig 11 for a comprehensive analysis. LYZL6 expression was detected in in the epididymis and testes and also in the head region of the spermatozoa (Fig 13). LYZL7 expression was undetectable in all the tissues analyzed (Fig 14).

### Muramidase assay

**Fig 15:**
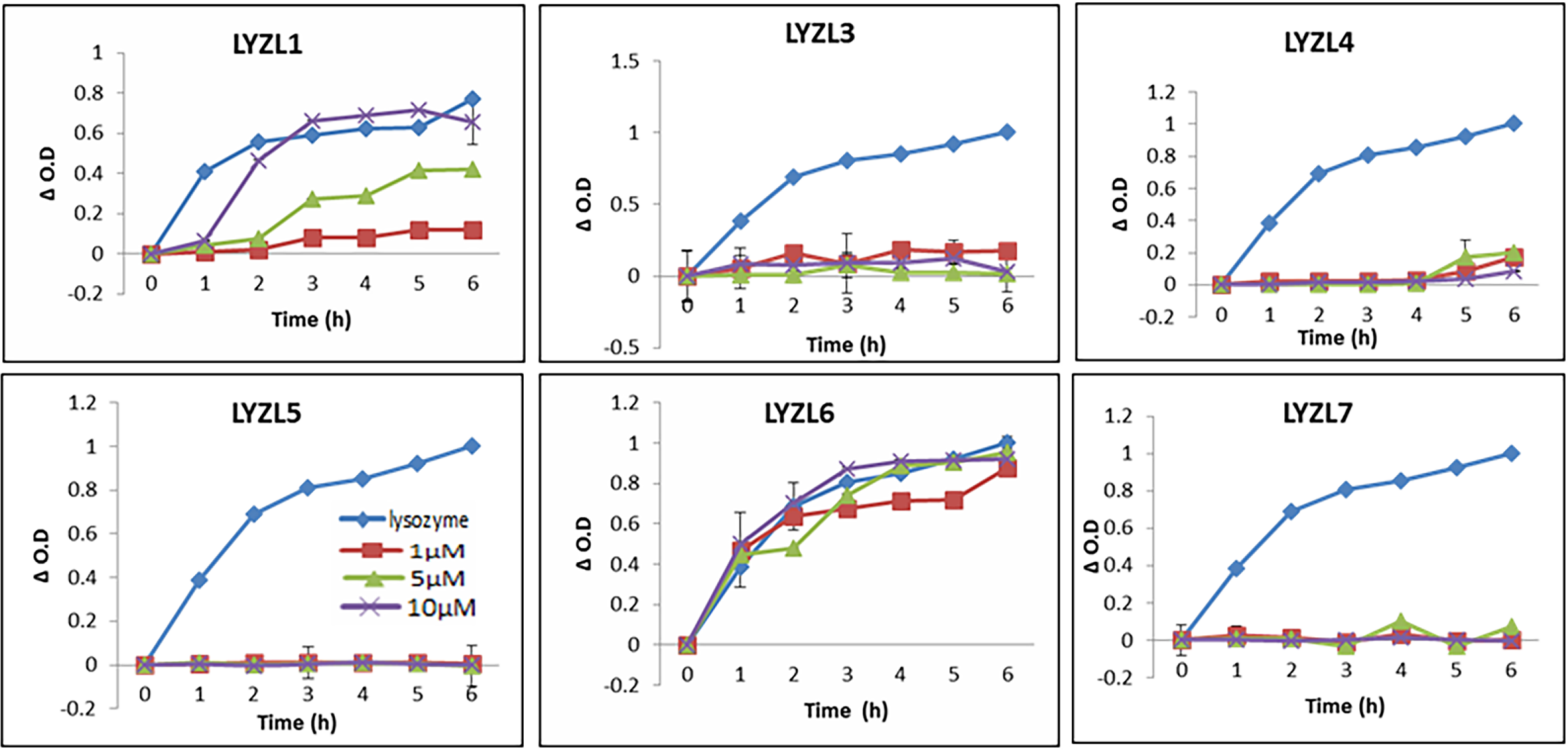
Muramidase activity of LYZL proteins. 1 μM (■), 5 μM (▲) and 10 μM (X) of recombinant LYZL proteins were incubated with 2 ml of *M*. *lysodeikticus* culture and the O.D monitored at 450nm. Chicken lysozyme (1μM; ♦) was used as a positive control. Values shown are mean ± SD.

Only LYZL1 and LYZL6 proteins exhibited a concentration dependent muramidase activity and was comparable to the positive control, lysozyme. The time course activity of LYZL6 was similar at all the concentrations used. This could be due to a very high activity of this protein even at the lowest concentration used. The remaining LYZL proteins did not show any muramidase activity (Fig 15) The activity of LYZL4 was previously reported by us [24] and the same result is included in Fig 15 for comparison.

### Isopeptidase assay

**Fig 16:**
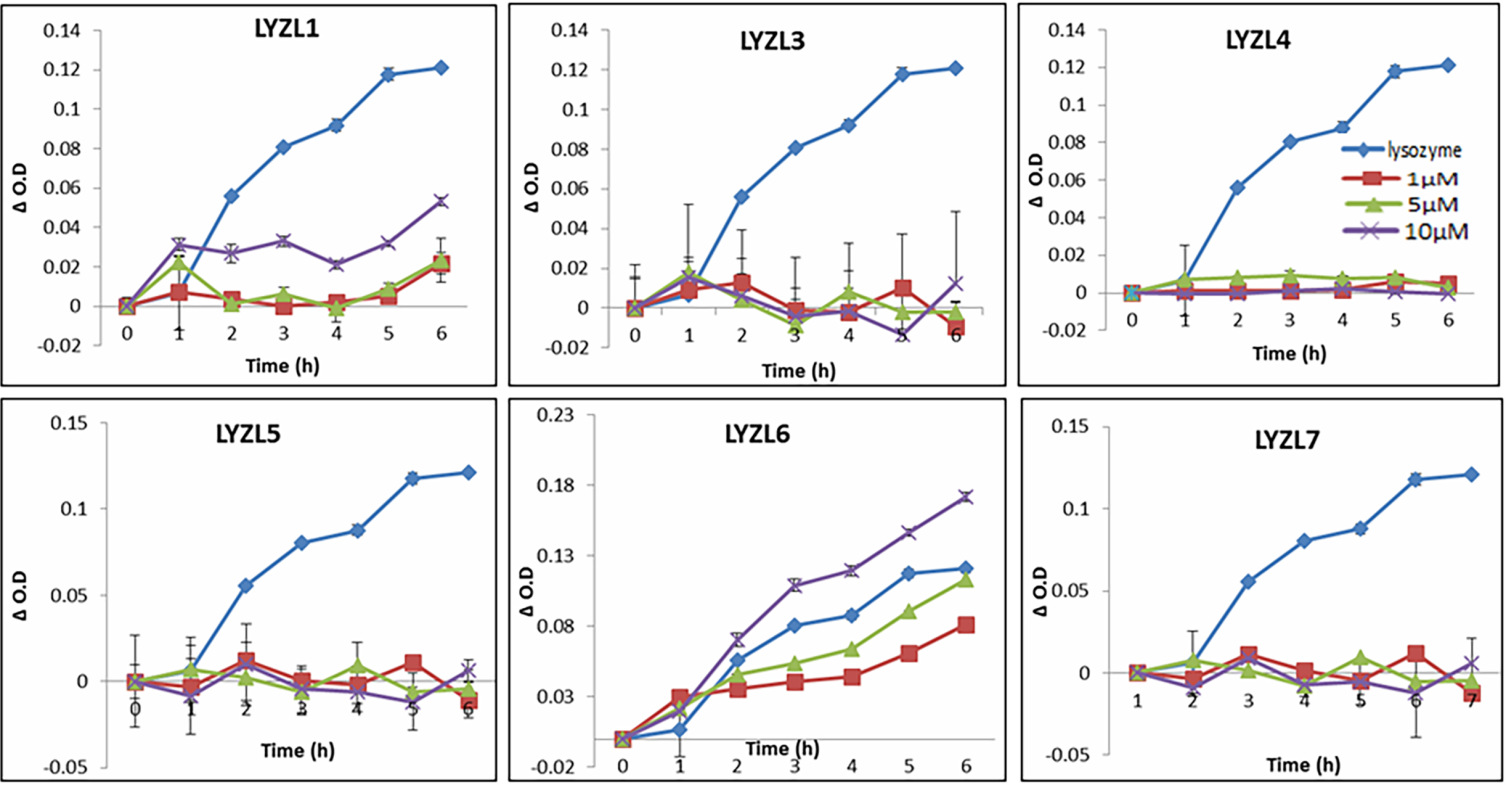
Isopeptidase activity of recombinant LYZL proteins. 1 μM (■), 5 μM (▲) and 10 μM (X) of recombinant LYZL proteins were incubated with 1.75 mM L-γ-Glu-pNA in 0.05 M 3-(N-morpholino) propane sulfonic acid (MOPS) buffer, pH 7, containing 0.01M NaCl and the O.D monitored at 405nm. Chicken lysozyme (1μM; ♦) was used as positive control. Values shown are mean ± SD. The isopeptidase activity of LYZL4 was reported by us earlier [24] and the same data is included in this figure for a comprehensive presentation of results.

Among the LYZL proteins that were tested, only LYZL1 and LYZL6 displayed isopeptidase activity in a concentration dependent manner, whereas LYZL3, 4, 5 and 7 did not exhibit any isopeptidase activity (Fig 16). We previously reported the activity of LYZL4 [24] and the same result is included in this figure for comparison.

### Antibacterial assay

**Fig 17:**
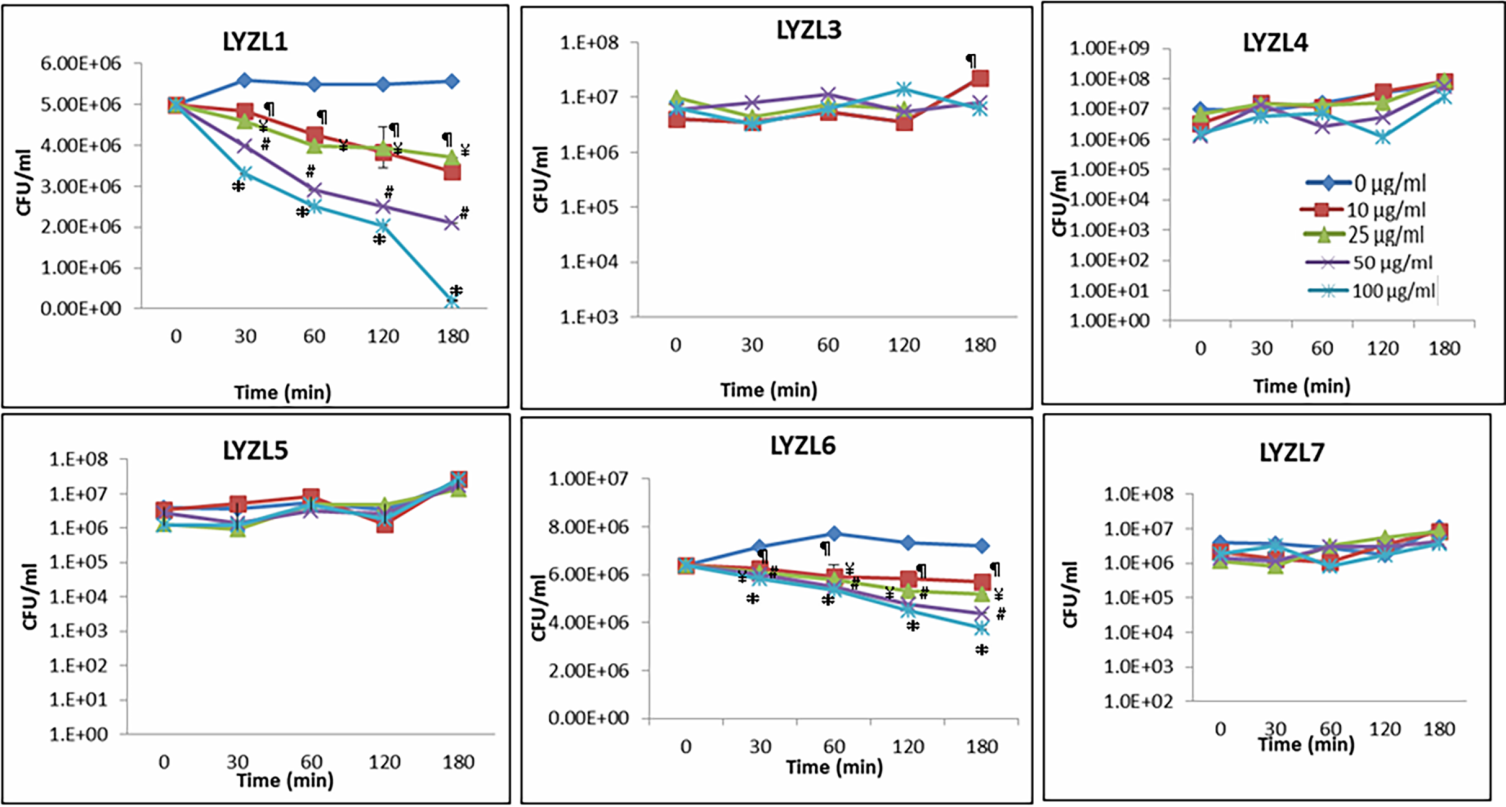
Antibacterial activity of LYZL proteins. *E*. *coli* XL-1 blue grown to mid-log phase were incubated with 0 (♦), 10 (■), 25 (▲), 50 (X) and 100 (*) μg/ml of recombinant protein for 30, 60, 90 and 120 min after the start of incubation. The assay mixtures were diluted, plated on LB agar plates and the colonies hand counted. Values shown are Mean ± S.D. The isopeptidase activity of LYZL4 was reported by us earlier [24] and the same data is included in this figure for a comprehensive presentation of results.

Colony forming units (CFU) assay was employed to test the antibacterial activity of LYZL proteins. LYZL1 and LYZL6 exhibited bacterial killing activity, whereas the remaining proteins failed to decrease bacterial count (Fig 17). This may be due to absence of the active site in these proteins. Antibacterial activity of LYZL4 was reported [24] and the same result is included in this figure for comparison.

### Measurement of membrane potential and Permeability

**Fig 18:**
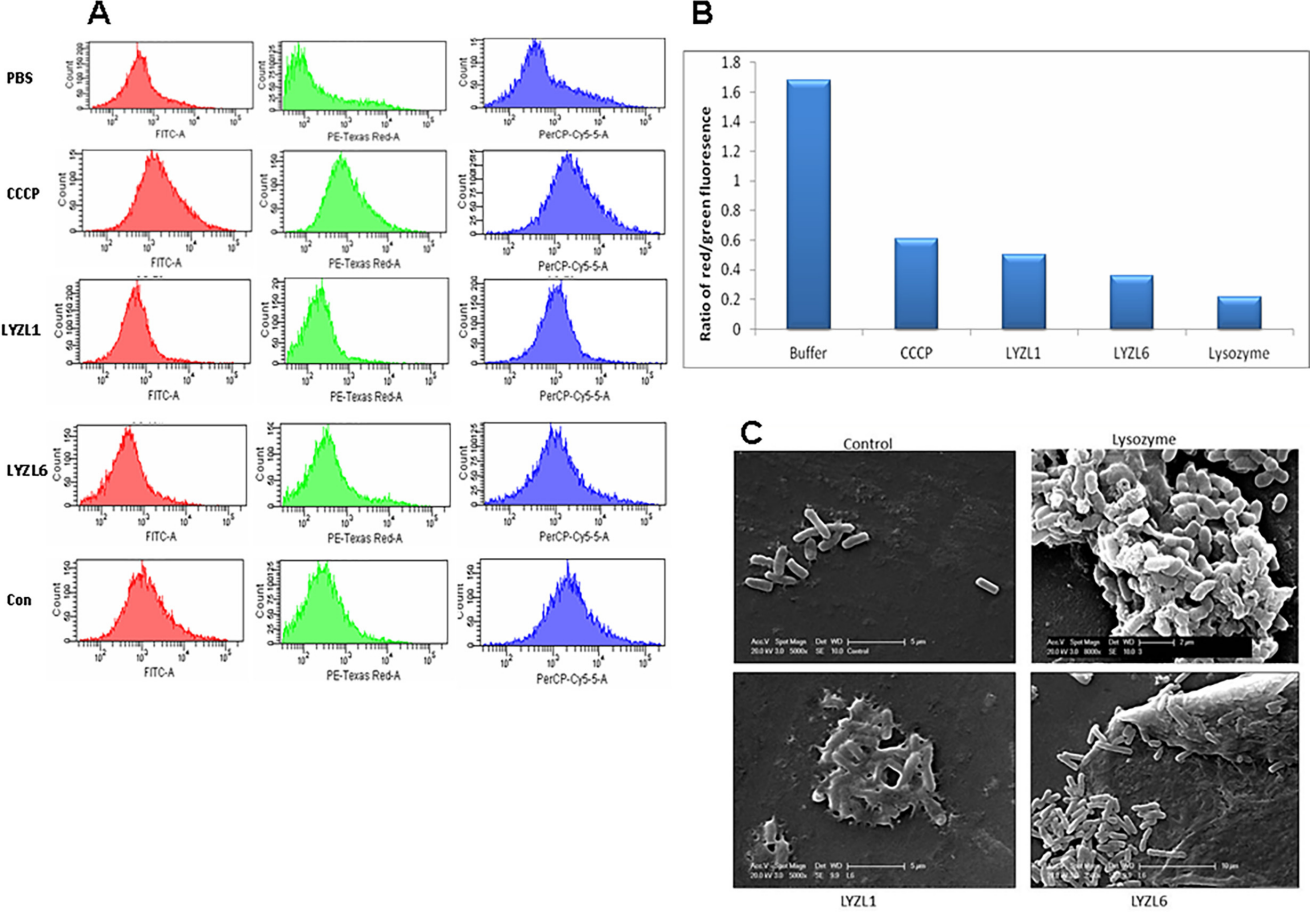
Membrane permeabilizing ability of LYZL proteins. **(A)** Dye based membrane potential measurement in *E*. *coli* treated with recombinant LYZL proteins using flow cytometry. A- MFI measurements of the cells in FITC, PE-Texas Red A and PerCP-Cy5-5-A channels. **(B)** Membrane potential measured in terms of ratio of mean fluorescence intensity of red/green. **(C)** Effect of recombinant LYZL1 and LYZL6 on the morphology of *E*. *coli*. Scanning electron micrographs of *E*. *coli* treated with 100 μg/ml recombinant LYZL proteins for 2 h. Chicken Lysozyme (10 μg/ml) was used as positive control.

The membrane potential and permeability of the bacterial cells treated with recombinant LYZL proteins was measured using DiOC_2_(3). Fig 18A shows the measurement of DiOC_2_(3) fluorescence in FITC-A (green) and PE-Texas Red-A (red) channel. The mean fluorescence intensity on PE Texas Red-A channels denotes the aggregation of the dye due to increased membrane potential. CCCP treatment caused decrease in membrane potential thereby decreased mean fluorescence intensity in PE-Texas Red-A channel. Green fluorescence is the measure of cell size to detect aggregation. CCCP treatment did not cause aggregation of bacterial cells which is indicated by the mean fluorescence intensity in FITC-A channel (Fig 18A). Treatment of cells with recombinant LYZL1 and 6 caused increase in green fluorescence showing that they possibly tend to aggregate bacterial cells (Fig 18A). Normalized ratio between the red and green fluorescence shows the membrane potential independent of cell size. Addition of recombinant LYZL1 or 6 protein to *E*. *coli* resulted in decreased ratio of red/green fluorescence in comparison to phosphate buffer treated bacterial cells (Fig 18B), suggesting clump formation and also change in membrane potential due to addition of these proteins. The bacterial cells treated with lysozyme also showed a change in the membrane potential similar to CCCP. TOPRO-3fluorescence is measured in PerCP-Cy5-5A channel. Increase in mean fluorescence intensity denotes increase in the membrane permeability. Treatment with LYZL proteins resulted in increased TOPRO-3 fluorescence indicating the ability of these proteins to cause membrane permeabilization (Fig 18A). *E*. *coli* treated with recombinant LYZL1 and LYZL6 were observed under electron microscope to study the morphological changes caused by these proteins. PBS treated *E*. *coli* cells show normal smooth surface (Fig 18C) whereas the LYZL1 and LYZL6 treated cells display rough cell surface with membrane blebbing. In addition, release of cytosolic content of the bacterial cells was observed. The actions of these proteins are similar to that exhibited by lysozyme.

### Peptidoglycan binding ability

**Fig 19:**
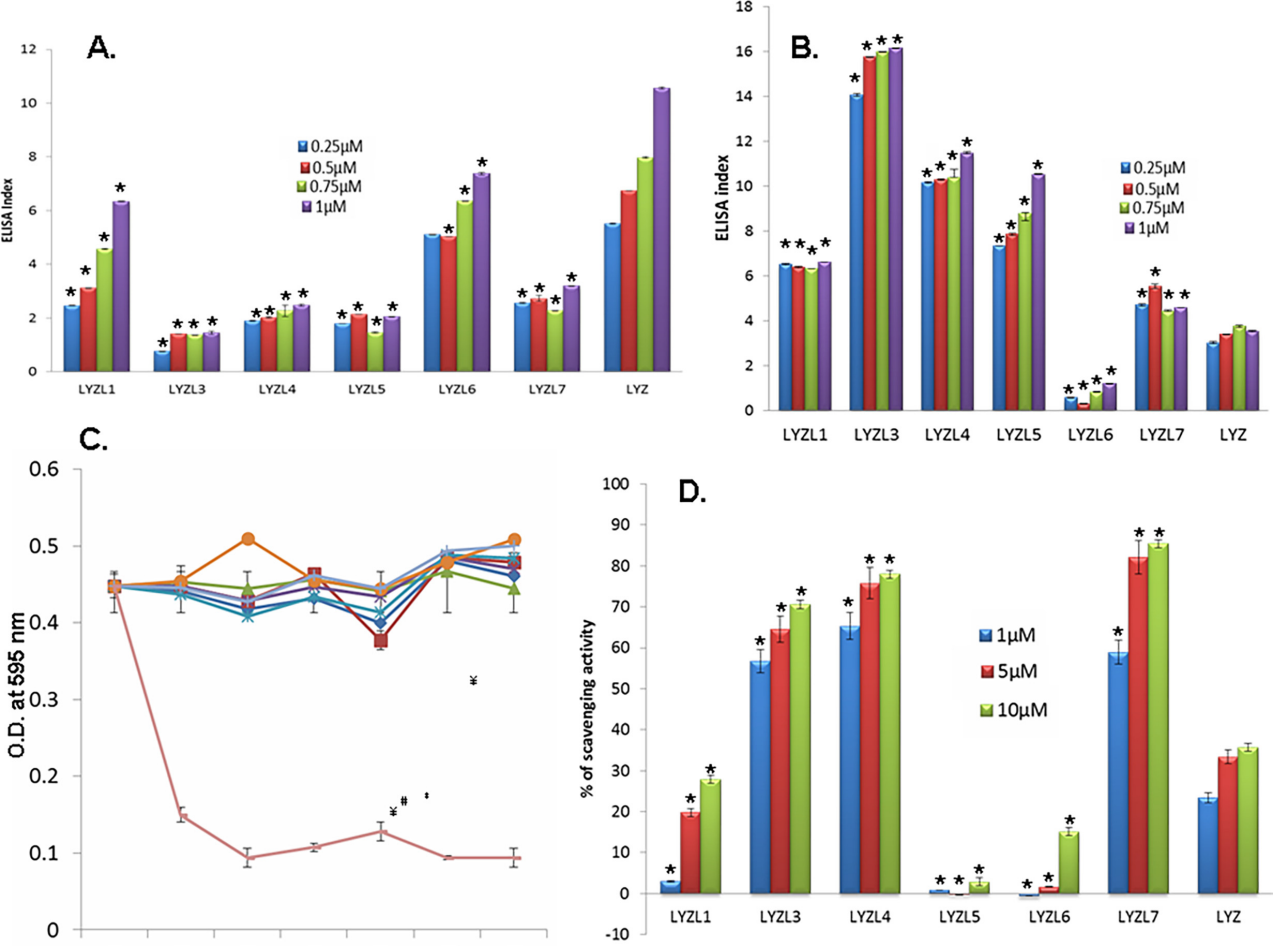
Substrate binding ability of LYZL proteins. **(A)** ELISA based peptidoglycan binding assay. 40 μg/ml peptidoglycan coated plate was incubated with 0.25, 0.5, 0.75 and 1μM of the recombinant proteins. Affinity of protein bound to peptidoglycan was measured in terms of color produced during development after probing with corresponding primary and secondary antibody. **(B)** ELISA based hyaluronan binding assay. 40 μg/ml hyaluronan coated into the wells of a microtitre plate was incubated with 0.25, 0.5, 0.75 and 1μM of the recombinant protein. Protein binding to peptidoglycan was measured by ELISA based colour detection. ELISA index was calculated by subtracting the average O.D of negative control and dividing the resultant by the negative control O.D. **(C)** Hyaluronidase activity of LYZL proteins. Hyaluronan mixed agarose incubated with different concentrations of recombinant LYZL proteins. The amount of cetyl pyridinium chloride precipitate cleared is a measure of hyaluronidase activity. Hyaluronidase was used as positive control. **(D)** Free radical scavenging assay. DPPH solution was incubated with varying concentrations of recombinant LYZL protein and the decrease in O.D was measured at 517 nm. Lysozyme was used a control. Radical scavenging activity was expressed in terms of percentage. Values shown are mean ± SD. * indicates p<0.05 compared to the corresponding concentration of lysozyme.

LYZL domain, which has the catalytic cleft and is responsible for binding to cell wall component, was found to be present in all the rat LYZL proteins. We observed that though all LYZL proteins possess the domain, only LYZL1 and LYZL6 show antimicrobial activity. Hence, analyzing the binding efficiency of the LYZL proteins with the bacterial cell wall components may help in understanding the differential antibacterial ability. As anticipated, lysozyme displayed a concentration dependent peptidoglycan binding ability (Fig 19A). LYZL1 and LYZL6 had higher peptidoglycan binding ability than LYZL3, 4, 5 and 7, which may be due to presence of active site. However, the binding ability of all the LYZL proteins was significantly less than lysozyme at all the concentrations tested (Fig 19A).

### Hyaluronan binding ability

Lysozyme exhibited hyaluronan binding in a dose dependent manner which may be due to chemical similarity between hyaluronan and peptidoglycan (Fig 19B). Among the LYZL proteins tested, LYZL3 had the highest hyaluronan binding ability followed by LYZL4, LYZL5, LYZL1 and LYZL7. LYZL6 had the least hyaluronan binding ability (Fig 19B).

### Hyaluronidase activity

Hyaluronidase used as a positive control caused the clearance of cetyl pyridinium chloride resulting in decrease of O.D at 595 nm. Surprisingly, though LYZL proteins had hyaluronan binding ability, none of them exhibited hyaluronidase activity at all the concentrations tested (Fig 19C).

### Free radical scavenging activity

Lysozyme exhibited potent free radical scavenging activity, which is evident by the discoloration of DPPH and there by decrease in the O.D at 517 nm. Except for LYZL5 and LYZL6, all other LYZL proteins caused a decrease in the O.D of DPPH at 517 nm in a dose dependent manner. Among them LYZL4 had the highest antioxidant potential (Fig 19D).

## Discussion

In this study we report the molecular characterization of rat LYZL proteins using *in silico* methods. The rat *Lyzl* genes were found to be located on different chromosomes suggesting that they are independent genes and are not isoforms coded by a single gene. All the LYZL proteins display similar physical properties such as molecular weight, pI and post translational modifications. Post translational modifications are crucial for proteins involved in the male reproductive tract function [44,45]. The specific posttranslational modifications that occur in LYZL proteins and their relevance to spermatogenesis, sperm maturation and function are yet to be investigated.

Similarity search using BLAST shows that rat LYZL proteins are similar to c-type lysozyme and conserve the characteristic lysozyme-like super family domain and the 8 cysteines that form four disulfide bonds. The catalytic mechanism of c-type lysozymes involves the interaction of Glu35 and Asp52 of the active site with beta-1,4 glycosidic bond of the substrate [46]. Both the active site amino acid residues are conserved only in LYZL1 and LYZL6, whereas they are partially conserved in LYZL4 and LYZL5 and completely absent in LYZL3 and LYZL7. However, all the LYZL proteins contain the additional substrate binding sites similar to c-type lysozyme. These observations suggest that LYZL proteins could have arisen from single gene and diverged at a later time point. LYZL7 is slightly different from the remaining LYZL proteins, in terms of similarity and identity with lysozyme and the presence of calcium binding site, which is a not feature of lactalbumin. At the secondary and tertiary structure levels, LYZL proteins predominantly contain α-helical pattern, a feature similar to lysozyme [47]. Docking studies indicate substrate interactions of LYZL 1 and 6 and the amino acids present in the binding site of all LYZL proteins are characteristic to lysozyme. Amphipathicity is an important feature of antimicrobial proteins [48]. All the rat LYZL proteins identified in this study are amphipathic in nature. However, the antimicrobial nature of rat LYZL proteins seem to be predominantly determined by the presence of the two active site amino acid residues. The conservation of active site amino acids in LYZL1 and LYZL6 confers them antimicrobial activity and may play a role in reproductive tract immunity. On the other hand, the partial conservation or absence of active site amino acid residues and the dissimilarity of LYZL7 indicates a divergence in the functional role in the reproductive tract of these proteins belonging to the same family.

Identification and functional characterization of testicular and epididymal proteins provided insights in to the molecular and physiological mechanisms in male reproduction [49–51] and continues to be an active area of research. In order to determine the role of LYZL proteins in male reproduction in this study, we report the expression pattern of two additional rat *Lyzl* transcripts and proteins and present the results in combination with those observed for other *Lyzl* genes characterized by us in a previously [24]. Some of the *Lyzl* mRNA transcripts were found to be expressed only in testes in the male reproductive tract. Such an exclusive expression in the testis suggests a role for these proteins in spermatogenesis. In addition to their expression in testis LYZL1, 4 and 7 are expressed in other non-reproductive tissues suggesting that these proteins may have roles beyond reproduction. Human LYZL4 transcripts were detected in the testes and pancreas similar to the expression pattern observed in this study [16]. The expression of mouse *Lyzl* genes in testes and epididymis was also reported [19]. Further LYZL4 was detected in brain and lungs in addition to testes and epididymis in mouse [23]. Our results indicate the expression of *Lyzl*4 in brain, lungs, kidney, ovary and uterus in addition of testes and epididymis. These results are more or less similar to that observed in earlier reports. However there are variations in the tissue expression pattern of these genes in different species indicating a possible variation in functional role in different species. On the other hand, an ortholog of the human LYZL2 gene is not found in the rats. The *Lysc1* gene that codes for calcium binding lysozyme is present only in higher mammals and not in rodents and this was due to deletion of the genomic region during evolution [52]. It is possible that the genomic region that contains the *Lyzl2* gene in the rat could have been deleted during evolution.

Developmental regulation of a wide variety of genes due to the fluctuations of androgens at various stages in the male reproductive system has been studied extensively [53]. Androgen levels in the testis, epididymis and blood vary during development in rodents [23,54]. *Lyzl* mRNA transcripts were not detected in the epididymides obtained from 20–60 day old rats suggesting that their expression pattern is not androgen dependent in this organ system. The presence of *Lyzl1*, *3*, *4*, *5*, *6* and *7* mRNA transcripts was observed in the testes starting from 30 day post-natal development and seem to correlate with the minimal androgen levels suggesting that *Lyzl* gene expression may be androgen dependent during development in the testis. Androgen dependent expression of *Lyzl4* during development was reported in the mouse testis [23]. Further studies are required to determine the molecular mechanisms that operate in controlling the expression of *Lyzl* transcripts during development.

Though the mRNA expression pattern of *Lyzl* genes are reported, this is the first study to report the LYZL protein expression pattern in male reproductive tract by immunolocalization and Western blotting. Localization of LYZL proteins in growing spermatids and in the germinal epithelium indicates that they may be secreted and added on to the sperm surface during spermatogenesis. LYZL4 was found to be localized in tail portion of mouse spermatozoa [19]. Similar observation was made in case of LYZL6 [23]. Our results are in consistent with earlier reports. Though some of the Lyzl gene expression was testis specific, their protein products were detected in epididymis also. This could be due to the movement of these proteins along with luminal fluid from testis to epididymis. The localization of LYZL proteins on the sperm surface indicates their possible role in spermatogenesis, sperm maturation, capacitation and acrosome reaction, sperm-egg fusion and fertilization. Studies using active immunization or knock out models will pin point the role of each of these proteins in the male reproductive tract.

Some of the proteins in the testicular and epididymal mileu bind to the spermatozoa and influence their function at multiple levels. Further, generation and maturation of male germ cells are regulated by the epididymal and testicular proteins [50,51]. Besides their role in sperm maturation and function, some of these proteins are known to exhibit potent antimicrobial activity, thereby forming important components of male reproductive tract innate immunity as well. Proteins belonging to the HE2 and PATE families exhibit potent antimicrobial activity, besides their role in sperm maturation and function [9,39,55]. Lysozyme is one of the abundant proteins in reproductive tract tissue secretions. Because of its ability to cleave the glycosidic bond of peptidoglycan, it displays potent antimicrobial activity [47] and also influences the microenvironment by regulating the viscosity of the semen [56]. Since LYZL proteins are similar to lysozyme, they may also play a role in male reproductive immunity. We observed that only LYZL1 and 6 display potent antibacterial activity against *E*. *coli*. The antibacterial activity of LYZL proteins was demonstrated in other species. For example, human LYZL6 was found to be a potent antibacterial protein [23]. LYZL3, 4, 5 and 7 did not display antibacterial activity. This could be due to the lack of essential amino acids in the active site. The human c-type lysozyme SLLP1, was non-bacteriolytic similar to rat LYZL3, 4, 5 and 7 [17,18]. Substrate binding assays also indicate that only LYZL1 and 6 exhibit higher affinity to bind peptidoglycan in comparison with the remaining proteins. Similar trend was observed in muramidase and isopeptidase assays. These properties can be attributed to the presence of active site residues in LYZL1 and 6. The antibacterial activity of LYZL1 and LYZL6 may play a significant role in male reproductive tract innate immunity. The lack of antibacterial activity of LYZL3, 4, 5 and 7 indicate that their role may be confined only to sperm function, whereas a broader role for LYZL1 and 6 may be expected. LYZL1 and 6 exhibited peptidoglycan binding ability, whereas the other LYZL proteins did not. The ability of LYZL1 and 6 to bind peptidoglycan could also contribute to their antibacterial activity. It is very interesting to speculate that LYZL proteins that do not conserve the active site amino acids and lack peptidoglycan binding ability may have evolved to perform another distinct set of functions that govern sperm function. On the other hand, LYZL3, 4, 5 and 7 proteins displayed potent hyaluronan binding ability than LYZL1 and LYZL6. Surprisingly, none of the LYZL proteins had hyaluronidase activity. Hyaluronan binding proteins play a crucial role in fertilization [57]. For example, the hyaluronan-binding protein modulates sperm-egg interaction and undergoes extensive phosphorylation, but lacks hyaluronidase activity [57]. The cellular events that occur during fertilization due to hyaluronin binding ability of LYZL proteins remains to be investigated.

Oxidative stress is a common factor in testicular dysfunction [58]. Antioxidant systems such as enzymes, metals and molecules and proteins exist in the testes to maintain the oxidant balance [59]. The free radical scavenging ability of LYZL proteins indicate a possible function for them in maintaining oxidative stress in the male reproductive tract. The free radical scavenging activity of lysozymes is attributed to the disulfide bonds in these proteins [60]. Though all LYZL proteins have four disulfide bonds, they vary in their ability of scavenge free radicals. The variation observed strengthens the possibility of diverse roles of these proteins though they belong to same family.

In conclusion, we report that rat LYZL proteins are predominantly expressed in the male reproductive tract. Their antibacterial activity, hyaluronin binding ability and free radical scanvenging activity indicate a diverse role for these proteins in male reproduction and immunity.

## Supporting Information

S1 Fig Flow chart of the docking anlyses. GOLD (Genetic Optimization for Ligand Docking) program was used to analyse the binding ability of LYZL proteins to N-acetyl glucosamine (NAG) trisaccharide.
(TIF)

S2 Fig Western blot analysis for cross reactivity. Recombinant LYZL proteins were separated and transferred on to nitrocellulose membranes. The immunoblots were probed with antibodies against each of the LYZL protein. L1 –LYZL1, L3 –LYZL3; L4 –LYZL4; L5 –LYZL5; L6 –LYZL6; L7 –LYZL7.
(TIF)

S3 Fig A neighbor joining phylogenetic tree for LYZL1 protein along with colour map, to show its conservation and distribution across the animal kingdom. (TIF)

S4 Fig A neighbor joining phylogenetic tree for LYZL3 protein along with colour map, to show its conservation and distribution across the animal kingdom. (TIF)

S5 Fig A neighbor joining phylogenetic tree for LYZL4 protein along with colour map, to show its conservation and distribution across the animal kingdom. (TIF)

S6 Fig A neighbor joining phylogenetic tree for LYZL5 protein along with colour map, to show its conservation and distribution across the animal kingdom. (TIF)

S7 Fig A neighbor joining phylogenetic tree for LYZL6 protein along with colour map, to show its conservation and distribution across the animal kingdom. (TIF)

S8 Fig A neighbor joining phylogenetic tree for LYZL7 protein along with colour map, to show its conservation and distribution across the animal kingdom. (TIF)

S1 Table Computational tools used for the *in silico* analyses of LYZL proteins in this study. (DOC)

S2 Table Gene specific primers used in this study. (DOC)
