# Supplementary figures and images for: In Silico and Biochemical Characterization of Lysozyme-Like Proteins in the Rat
Source: PLoS One. 2016 Sep 9;11(9):e0161909. doi: 10.1371/journal.pone.0161909 (PMC5017655; doi:10.1371/journal.pone.0161909)

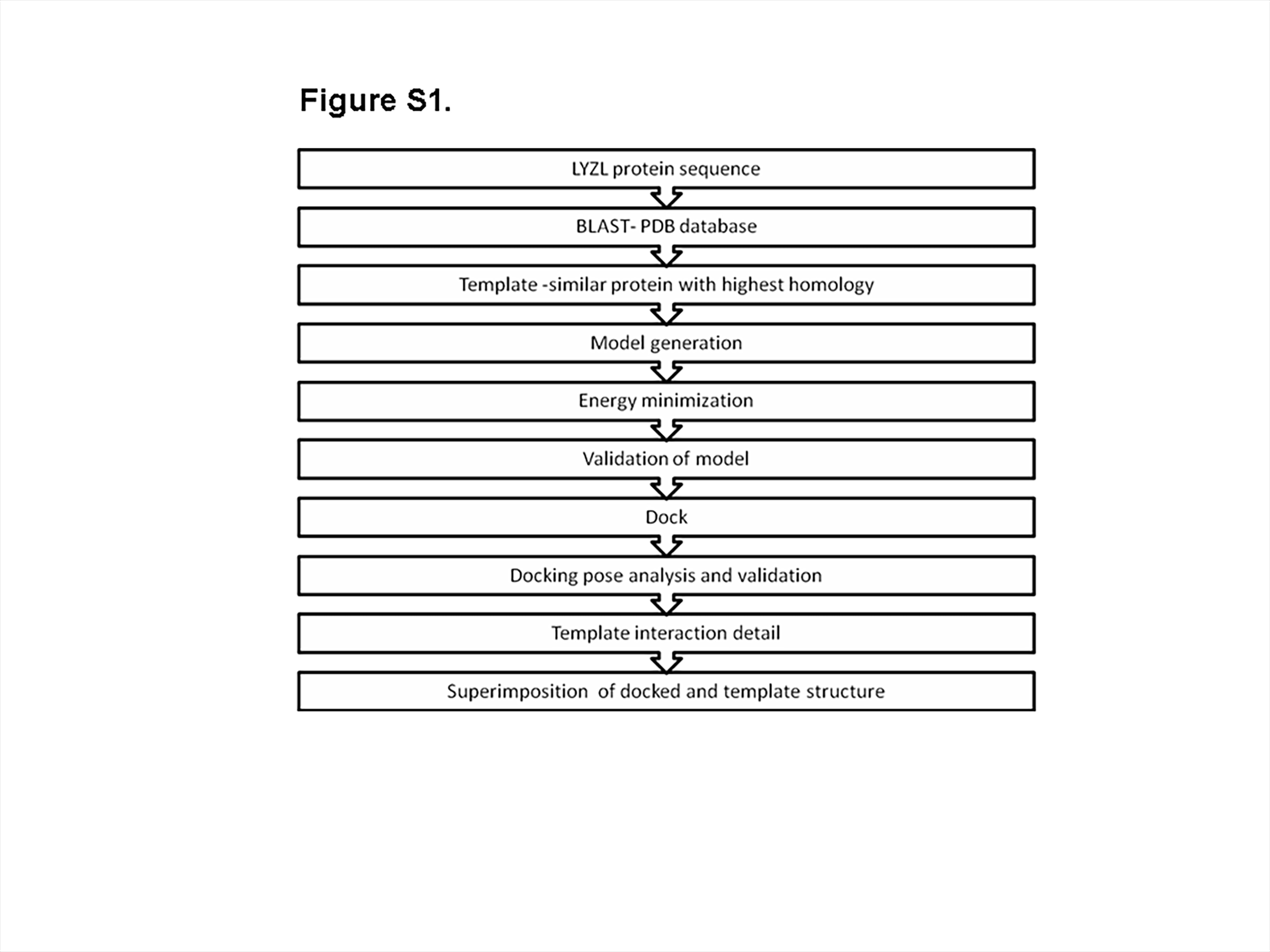

Supplement: S1 Fig — GOLD (Genetic Optimization for Ligand Docking) program was used to analyse the binding ability of LYZL proteins to N-acetyl glucosamine (NAG) trisaccharide. (TIF) [file pone.0161909.s001.TIF]

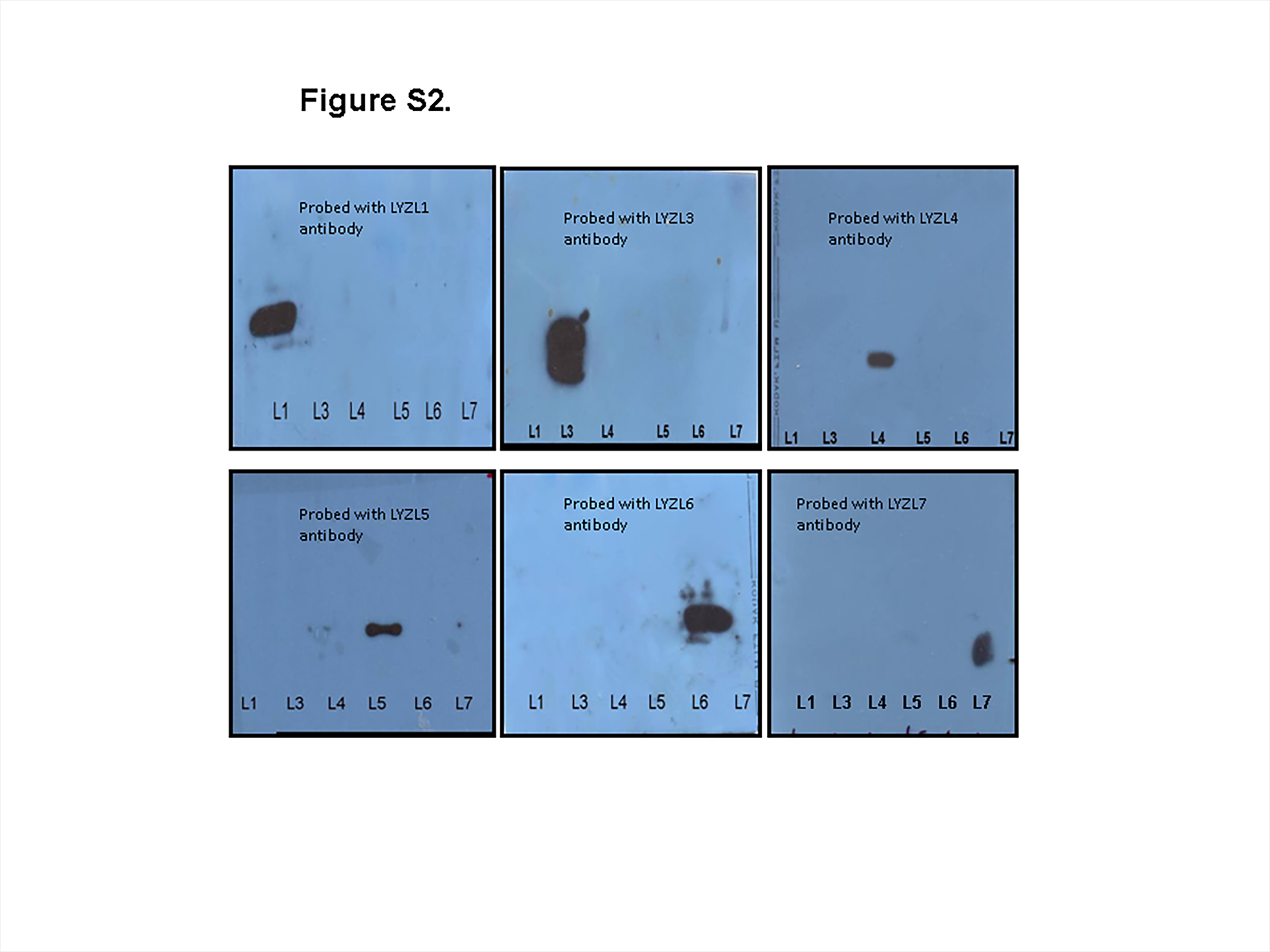

Supplement: S2 Fig — Recombinant LYZL proteins were separated and transferred on to nitrocellulose membranes. The immunoblots were probed with antibodies against each of the LYZL protein. L1 –LYZL1, L3 –LYZL3; L4 –LYZL4; L5 –LYZL5; L6 –LYZL6; L7 –LYZL7. (TIF) [file pone.0161909.s002.TIF]

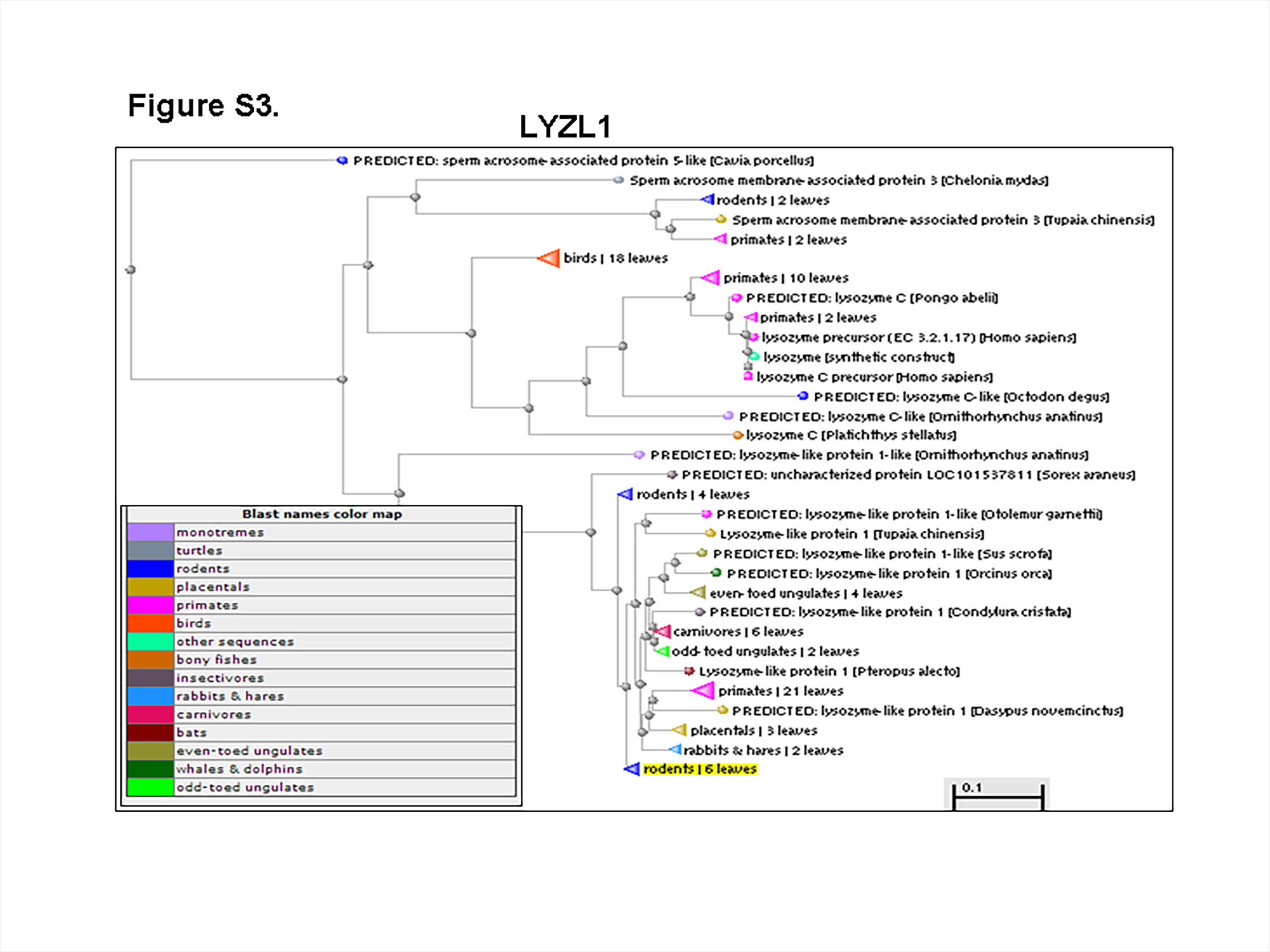

Supplement: S3 Fig — (TIF) [file pone.0161909.s003.TIF]

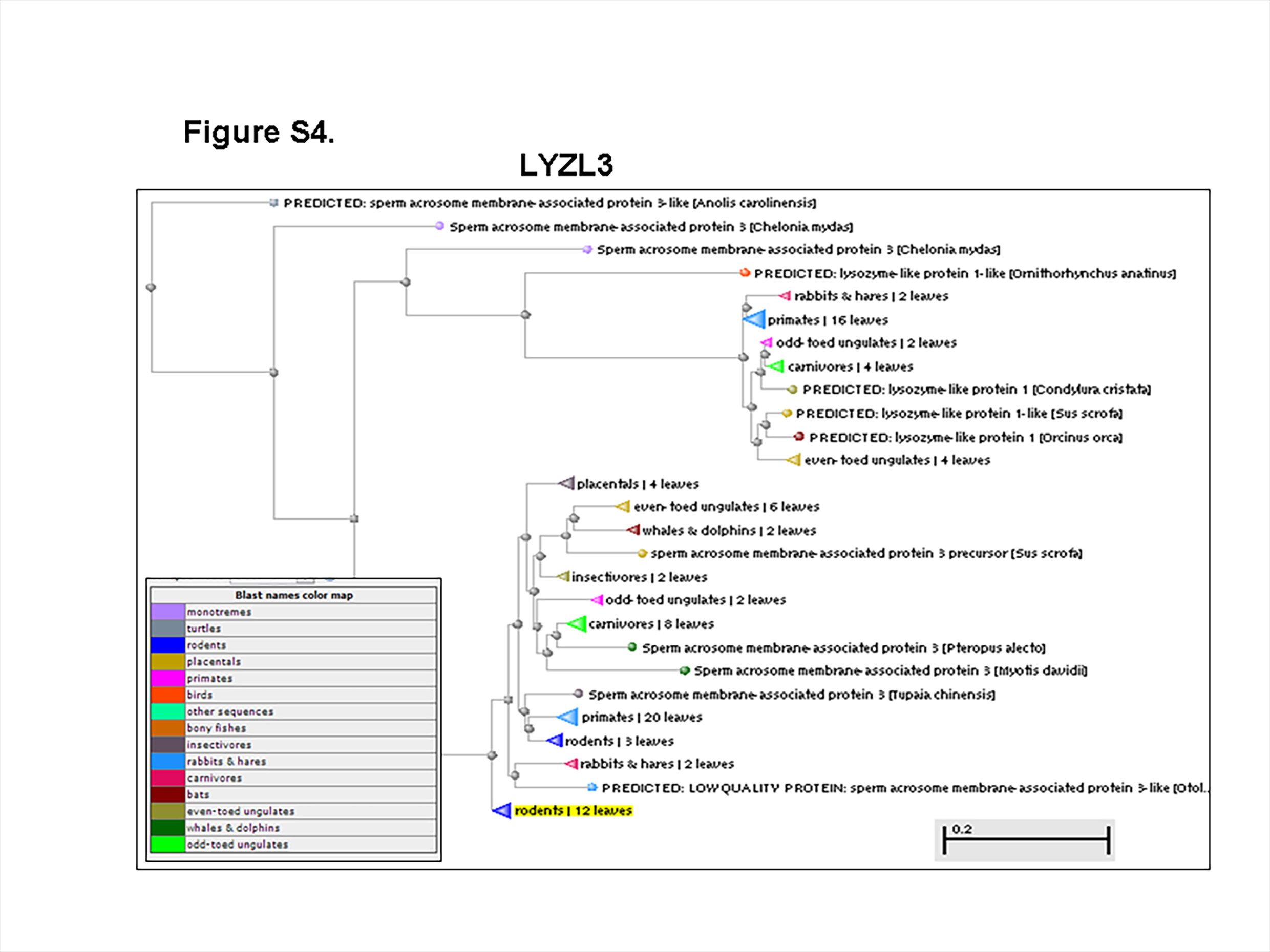

Supplement: S4 Fig — (TIF) [file pone.0161909.s004.TIF]

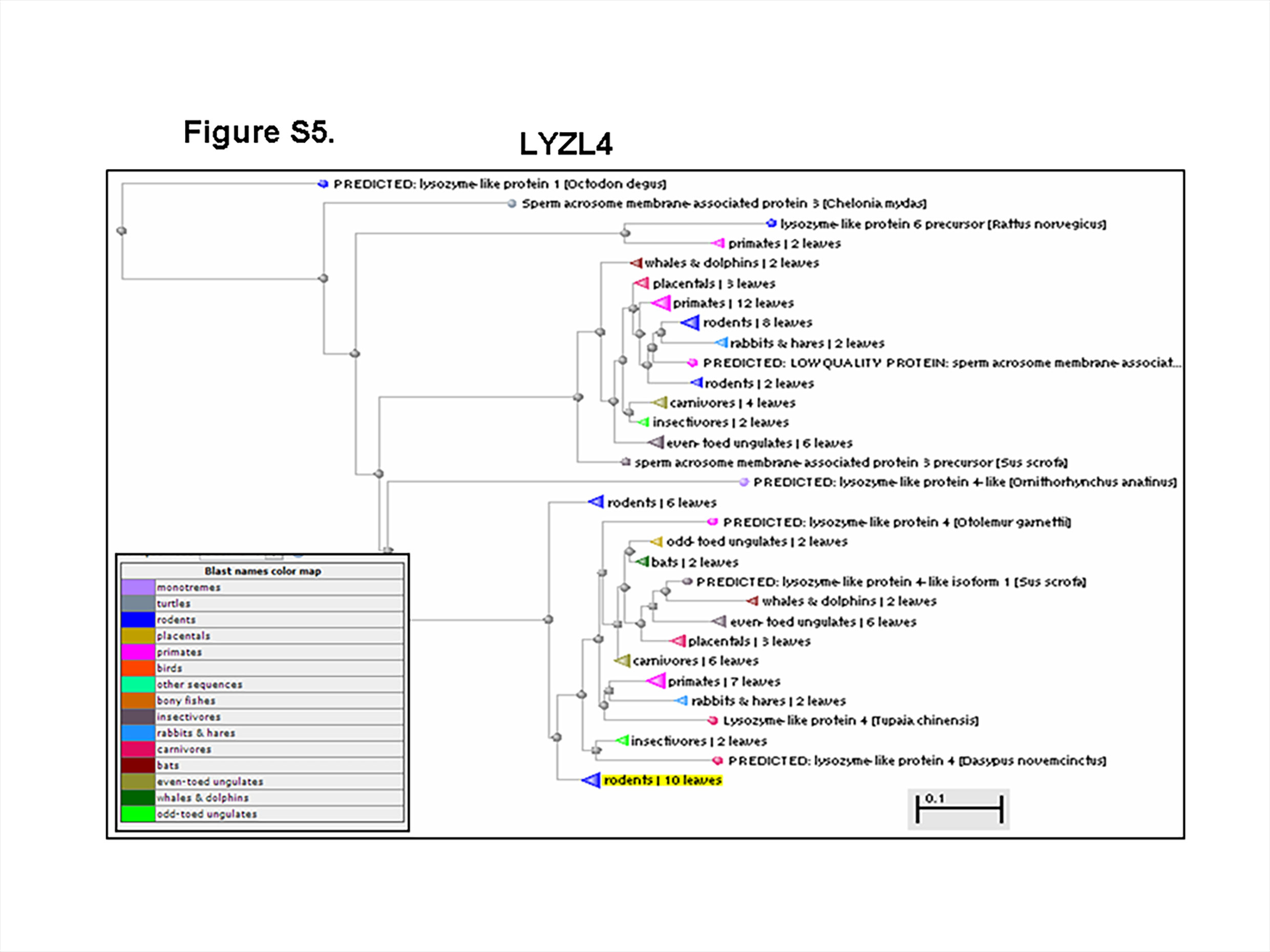

Supplement: S5 Fig — (TIF) [file pone.0161909.s005.TIF]

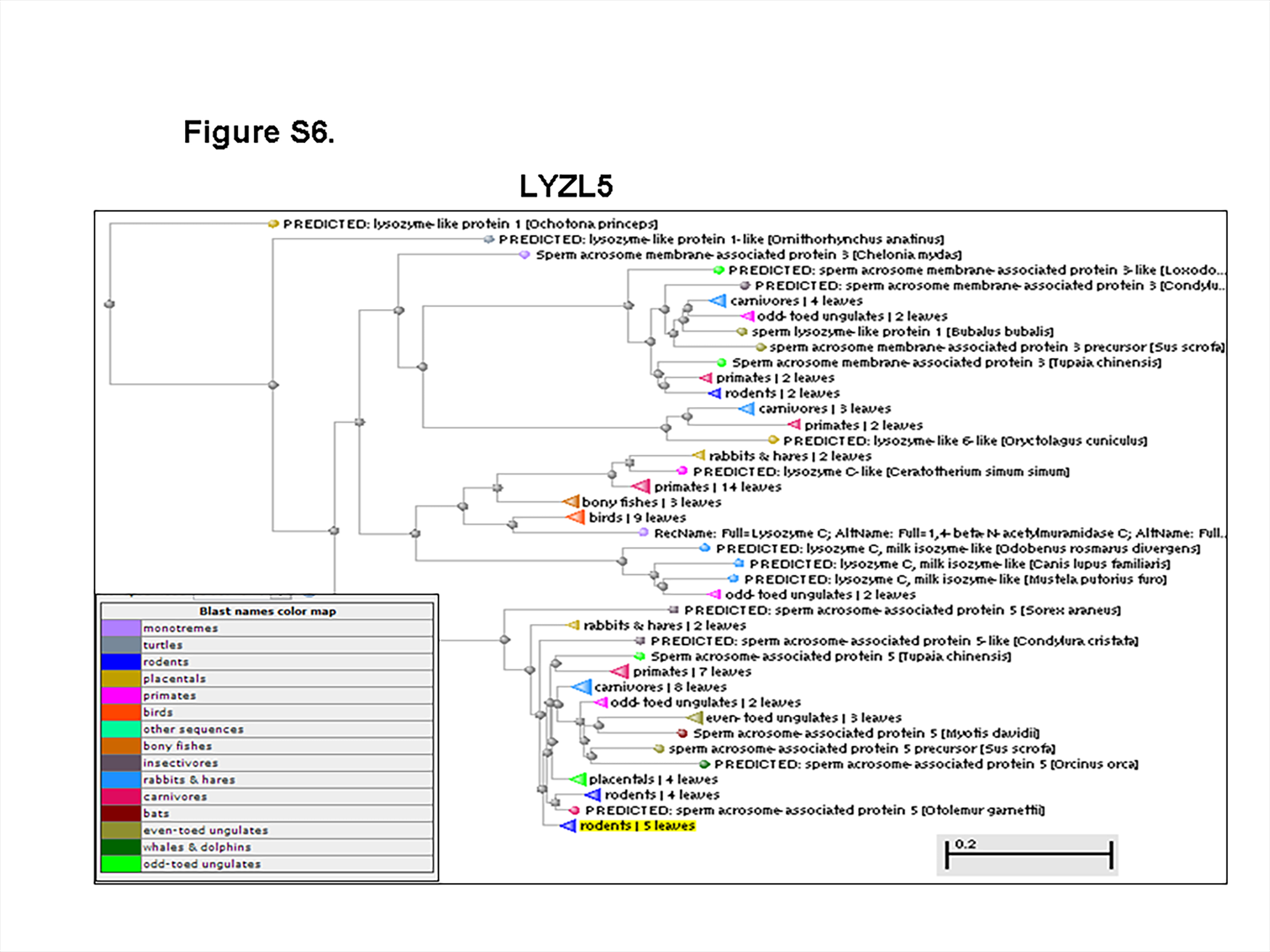

Supplement: S6 Fig — (TIF) [file pone.0161909.s006.TIF]

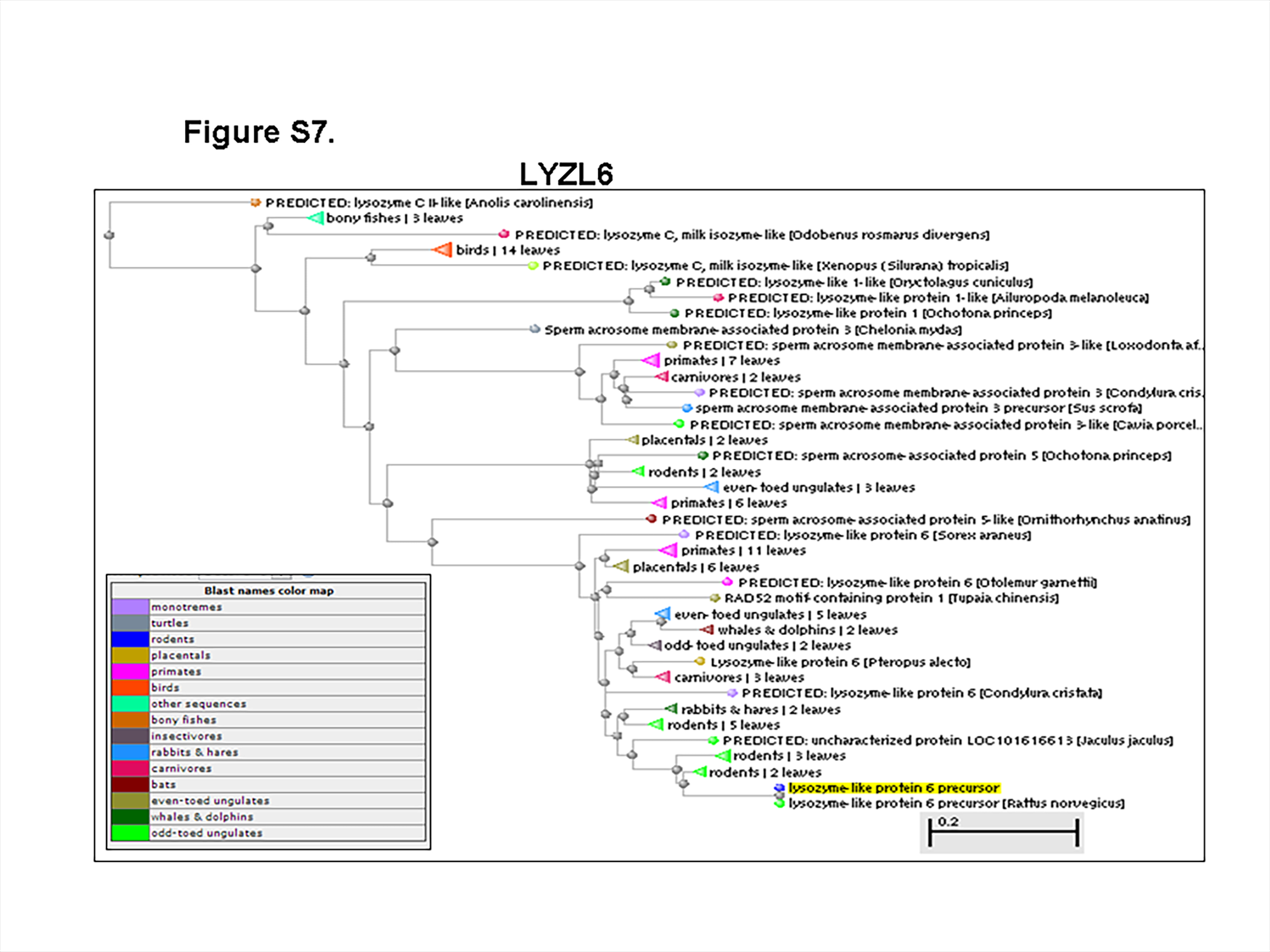

Supplement: S7 Fig — (TIF) [file pone.0161909.s007.TIF]

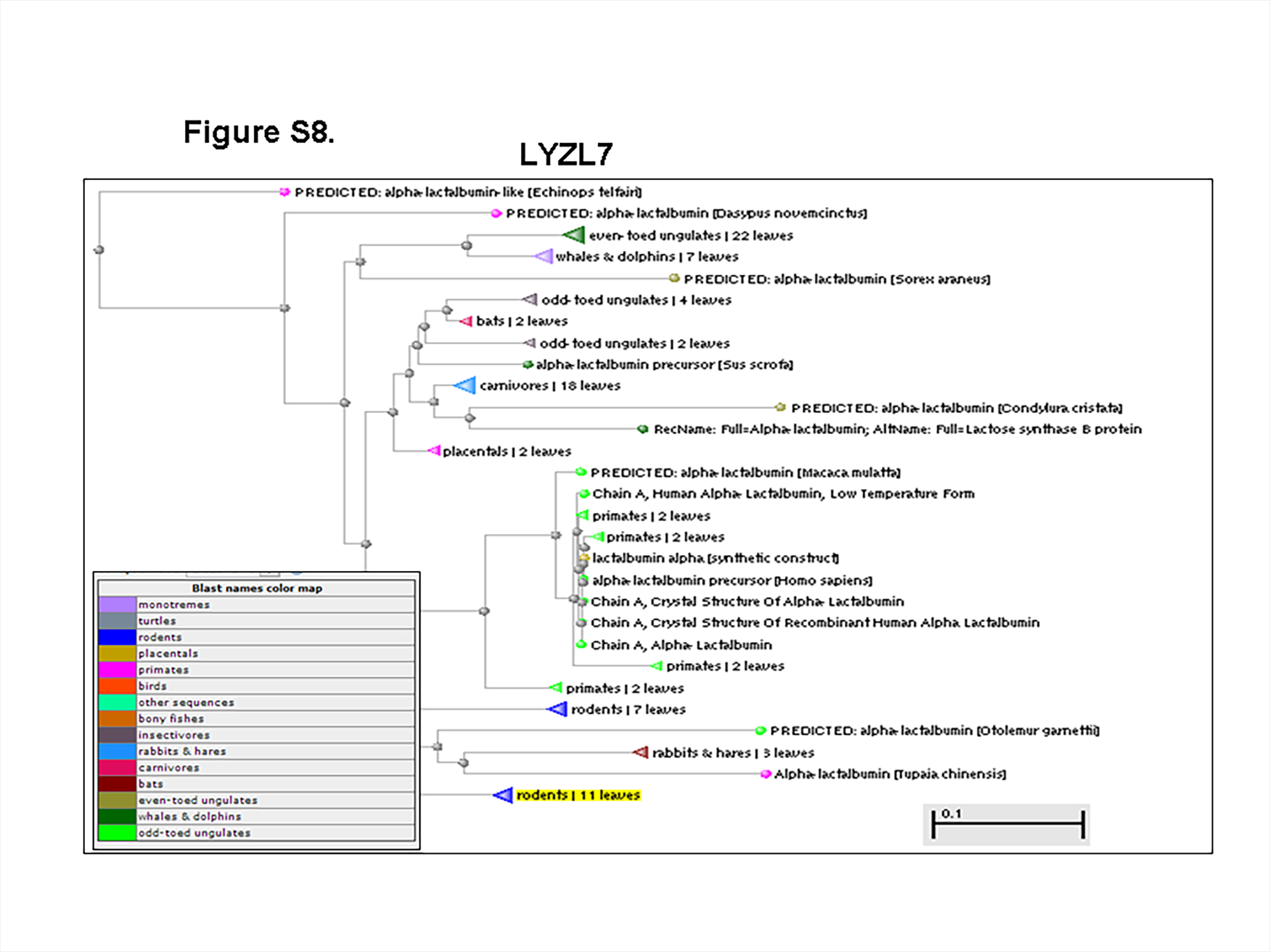

Supplement: S8 Fig — (TIF) [file pone.0161909.s008.TIF]
