# Supplementary material for: In Silico and Biochemical Characterization of Lysozyme-Like Proteins in the Rat
Source: PLoS One. 2016 Sep 9;11(9):e0161909. doi: 10.1371/journal.pone.0161909 (PMC5017655; doi:10.1371/journal.pone.0161909)
Supplement: S1 Table — (DOC) [file pone.0161909.s009.doc]

**S1 Table. Computational tools used for the *in silico*** analyses of LYZL proteins in this study.

| **Analysis** | **Tool used** | **Website** |
| --- | --- | --- |
| Sequence retrieval | NCBI nucleotide | http://www.ncbi.nlm.nih.gov/nuccore/ |
| Similarity search | NCBI BLAST | http://blast.ncbi.nlm.nih.gov/Blast.cgi |
| Multiple sequence alignment | T-COFFEE | http://tcoffee.crg.cat/ |
| Pairwise alignment | CLUSTALW | http://www.genome.jp/tools/clustalw/ |
| Conserved Domain prediction | NCBI CDD | http://www.ncbi.nlm.nih.gov/cdd/ |
| General properties | EXPASY server | http://expasy.org/proteomics |
| Post translational modification | Sequence manipulation suite | http://www.bioinformatics.org/sms2/ |
| Genomic neighbourhood | Ensembl | http://asia.ensembl.org/index.html |
| Phylogeny | BLAST | http://blast.ncbi.nlm.nih.gov/Blast.cgi |
