# Supplementary material for: In Silico and Biochemical Characterization of Lysozyme-Like Proteins in the Rat
Source: PLoS One. 2016 Sep 9;11(9):e0161909. doi: 10.1371/journal.pone.0161909 (PMC5017655; doi:10.1371/journal.pone.0161909)
Supplement: S2 Table — (DOC) [file pone.0161909.s010.doc]

**S2 Table 2.** Gene specific primers used in this study

| **Gene** | **Primer sequence (5’ --->3’)** | **No. of bases** | **Amplicon size (bp)** |
| --- | --- | --- | --- |
| *Lyzl1 FP* | TGTCGGTGTCTTCGCCCTAAT T | 22 | 408 |
| *Lyzl1 RP* | GAC GAG TCT TTG CTC TCA CAG T | 22 |  |
| *Lyzl3 FP* | TCC AGC AAG GCC AAG GTC TTC A | 22s | 398 |
| *Lyzl3 RP* | TAG AAG TCA CAG CCA TCC ACC CA | 23 |  |
| *Lyzl4 FP1* | ATG TGG GCA CTG TTG ACA CCA | 21 | 602 |
| *Lyzl4 RP1* | CTA CAC CAT TGA TCC TGC TCC A | 22 |  |
| *Lyzl5-FP* | CACGCATGCAAAGATTTATGAACGCTGTG | 29 | 420 |
| *Lyzl5-RP* | CAGGTCGACTCACCAGTCATCATAGT | 26 |  |
| *Lyzl6 FP* | TAT CTG TGT GGT GAG CTG CCT TCT | 24 | 322 |
| *Lyzl6 RP* | TGC ACA GTG GAT GGA TGGAAT GAG | 24 |  |
| *Lyzl7-FP* | TATGCATGCTACAGAGTTTACAAAATGTGA | 30 | 441 |
| *Lyzl7-RP* | AGGTCGACTTAGGGAACAGGTGTTTCTGAAT | 31 |  |

In our previous publication [24], we reported the primers used for some Lyzl genes and additional primers used along with those reported are included in this table.
